# Transcriptome analysis of the reef-building octocoral, *Heliopora coerulea*

**DOI:** 10.1038/s41598-018-26718-5

**Published:** 2018-05-30

**Authors:** Christine Guzman, Chuya Shinzato, Tsai-Ming Lu, Cecilia Conaco

**Affiliations:** 1 Marine Science Institute, College of Science, University of the Philippines, Diliman, Quezon City, 1101 Philippines; 2 Evolutionary Neurobiology Unit, Okinawa Institute of Science and Technology Graduate University, Onna, Okinawa, 904-0495 Japan; 3 Department of Marine Bioscience, Atmosphere and Ocean Research Institute, The University of Tokyo, Kashiwa-shi, Chiba, 277-8564 Japan; 4 Marine Genomics Unit, Okinawa Institute of Science and Technology Graduate University, Onna, Okinawa, 904-0495 Japan

## Abstract

The blue coral, *Heliopora coerulea*, is a reef-building octocoral that prefers shallow water and exhibits optimal growth at a temperature close to that which causes bleaching in scleractinian corals. To better understand the molecular mechanisms underlying its biology and ecology, we generated a reference transcriptome for *H. coerulea* using next-generation sequencing. Metatranscriptome assembly yielded 90,817 sequences of which 71% (64,610) could be annotated by comparison to public databases. The assembly included transcript sequences from both the coral host and its symbionts, which are related to the thermotolerant C3-Gulf ITS2 type *Symbiodinium*. Analysis of the blue coral transcriptome revealed enrichment of genes involved in stress response, including heat-shock proteins and antioxidants, as well as genes participating in signal transduction and stimulus response. Furthermore, the blue coral possesses homologs of biomineralization genes found in other corals and may use a biomineralization strategy similar to that of scleractinians to build its massive aragonite skeleton. These findings thus offer insights into the ecology of *H. coerulea* and suggest gene networks that may govern its interactions with its environment.

## Introduction

Octocorals are the most diverse coral group and can be found in various marine environments, including shallow tropical reefs, deep seamounts, and submarine canyons^1^. They reportedly exhibit greater resistance and resilience to environmental perturbations, particularly to thermal stress, compared to scleractinians^2–4^. They are also suitable indicators of ecological disturbances, as they are generally fast-growing and have the ability to colonize open spaces. Furthermore, octocorals provide habitats for molluscs, polychaetes, fish, and other marine fauna^5^. Octocorals contribute to the structure of reef communities through the production of allelopathic compounds that drive interactions with other organisms^6,7^.

The Octocorallia comprises approximately 3,500 extant species and is traditionally divided into three genetically distinct clades – the order Alcyonacea (soft corals and gorgonians), the Pennatulacea (sea pens), and the Helioporacea (blue corals)^8^. The Helioporacea is unique, as its members produce calcified skeletons of crystalline aragonite, a convergent feature shared with scleractinians. The blue coral, *Heliopora coerulea*, is the sole extant member of the family Helioporidae. Its fibrous aragonite skeleton is characterized by a distinctive and permanent blue pigmentation resulting from iron salts^9^. The blue coral is a reef-building coral that superficially resembles many scleractinian corals in its gross morphology. Its colonies exhibit considerable morphological plasticity and can be arborescent or plate-like. In very calm water it can form slender columns or in more turbulent areas, it develops stout trunks and branches. *H. coerulea* frequently co-exists with massive stony corals like *Porites* and *Montipora*^10^.

Based on fossil records, the genus *Heliopora* was widely distributed in shallow waters of all oceans^10^. However, climatic changes, such as glacial periods of lowered temperatures, may have resulted in contraction of its distribution. Presently, *H. coerulea* is found in equatorial waters between 25°N and 25°S in the Indo-Western Pacific region, Red Sea, American Samoa, southern Japan, and Australia. Although it is uncommon throughout its range, it is the dominant species on some reefs^10^. The distribution of *H. coerulea* may be determined by its optimal growth temperature, short larval duration, and other environmental factors. *H. coerulea* thrives in waters with a mean annual minimum temperature above 22 °C, which is considerably higher than the 18 °C marginal isotherm for many corals^10^. Its shallow depth range and higher optimal growth temperature are close to conditions that cause bleaching in scleractinian corals^11^.

Scleractinians, or hard corals, are the primary bioconstructors of reef ecosystems. However, in recent years, heat stress due to global warming and increasing intensity of El Niño Southern Oscillation (ENSO) events have worsened the bleaching of hard corals worldwide. Such recurrent disturbances have caused mass die-offs of many hard coral species, reducing their abundance and total cover^12^. Modification of reef substrata may allow opportunistic proliferation of non-scleractinian taxa, such as octocorals^12^. In fact, in some ecosystems, octocoral abundance is reported to be increasing coincidentally with scleractinian decline, following severe bleaching events^13,14^. More specifically, a shift in the dominant taxa from branching scleractinian corals to *H. coerulea* have been observed on reefs in Ishigaki island, Okinawa, Japan and Bolinao, Pangasinan, Philippines^15,16^.

To date, very few studies have examined octocorals and available transcriptomic data mostly represent corals of the order Alcyonacea^17,18^. Sequencing of the first octocoral transcriptome from the Carribean sea fan, *Gorgonia ventalina*, revealed mechanisms of sea fan immunity in response to pathogen exposure^18^, while a study of the red coral, *Corallium rubrum*, further highlighted the molecular basis of local adaptation to thermal stress^17^. Thus, while a few studies have begun to explain the stress tolerance and adaptive potential of some octocorals to environmental perturbations, transcriptome sequencing of other octocoral species will prove valuable in understanding the potential impacts of changing environments on members of this diverse coral group.

**Figure 1:**
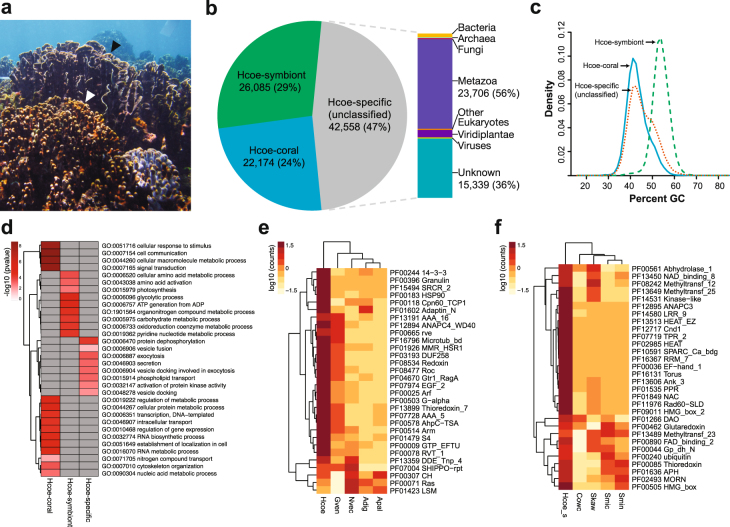
De novo transcriptome assembly of the blue coral, *Heliopora coerulea*. (**a**) *H. coerulea* contributes to reef structure through production of an aragonite skeleton that forms colonies with digitate (white arrowhead) or laminar (black arrowhead) morphologies. (**b**) Classification of *H. coerulea* transcripts into those originating from the coral host (Hcoe-coral) or the symbiont (Hcoe-symbiont). Unclassified transcripts are potentially *Heliopora*-specific (Hcoe-specific) with about 56% aligning to metazoan sequences. (**c**) Percent GC of transcript sets classified by origin. (**d**) Gene ontology (GO) analysis for *H. coerulea* transcripts classified by origin. Enrichment p-values for selected terms are shown. Only GO terms with a p-value < 0.05 (Fisher’s exact test) were considered significantly enriched. (**e**–**f**) Top 30 enriched PFAM protein domains in (**e**) Hcoe-coral and (**f**) Hcoe-symbiont peptides compared to other organisms. Heatmaps were generated using pheatmap in R. Species names are abbreviated as follows: *H. coerulea* coral host *(Hcoe), G. ventalina (Gven), N. vectensis (Nvec), A. digitifera (Adig), A. pallida (Apal), H. coerulea* symbionts *(Hcoe_s), C. owczarzaki (Cowc), S. kawagutii (Skaw), S. microadriaticum (Smic)*, and *S. minutum (Smin)*.

In this study, we describe a reference transcriptome of the non-scleractinian reef-building coral, *H. coerulea* (Fig. 1a). Gene content analysis and comparison with other cnidarian sequences potentially reveal the molecular basis for unique characteristics of this coral, its symbiotic interactions, and responses to the environment. This transcriptome resource provides a platform for future investigations into the gene complement and gene expression dynamics of *H. coerulea*.

## Results

### RNA sequencing and transcriptome assembly

Sequencing of cDNA libraries from three individual colonies of *H. coerulea* yielded a total of 108.7 million paired-end reads (Supplementary Tables 1, 2), of which 82.8 million remained after adapter trimming and quality filtering. *De novo* transcriptome assembly yielded 231,515 transcripts (>300 bp), with an N50 of 1,106 bp. 155,859 (67%) transcripts were translated into peptides and 90,817 (39%) were retained after clustering similar sequences with CDHIT (Supplementary Table 2).

### Classification of transcripts

Like most scleractinian corals, the blue coral is a holobiont consisting of a cnidarian host and endosymbiotic dinoflagellates of the genus *Symbiodinium*. To identify sequences in the *H. coerulea* transcriptome that originate from either the coral host or its symbionts, transcripts were aligned to cnidarian and *Symbiodinium* databases using blastn (Supplementary Table 3). This recovered 22,174 (24%) sequences originating from the coral host (Hcoe-coral) and 26,085 (29%) originating from symbionts (Hcoe-symbiont) (Fig. 1b, Supplementary Table 4). About 47% (42,558 sequences) of the transcripts could not be classified, as they did not match any sequences in the cnidarian and symbiont databases. The latter set of sequences may include transcripts specific to the *H. coerulea* holobiont (Hcoe-specific).

Transcripts classified as originating from the coral (Hcoe-coral) exhibited a GC content of about 43% (Fig. 1c). This is similar to the GC content of other anthozoans, such as *Acropora digitifera* (39%), *Porites australiensis* (40%), and *Nematostella vectensis* (47%)^19–21^. On the other hand, transcripts classified as originating from the symbiont (Hcoe-symbiont) had a GC content of approximately 54%, which is similar to the GC content reported for *Symbiodinium microadriaticum* (50.5%) and considerably higher than the coding GC content of *S. minutum* (43.5%) and *S. kawagutii* (45.5%)^22^. The GC content of unclassified (Hcoe-specific) transcripts also peaked at around 45% supporting the idea that most of these originated from the blue coral host or other organisms associated with the coral holobiont.

### Transcriptome annotation

In total, 64,610 (71%) of the 90,817 *H. coerulea* transcriptome sequences were annotated using public databases (NCBI RefSeq, UniProt-SwissProt, PFAM, and KEGG) (Supplementary Table 5). Specifically, blastp alignments revealed that 61,922 (68%) and 49,796 (55%) had significant matches to proteins in the NCBI-RefSeq and UniProt-SwissProt databases, respectively. A total of 21,573 (97%) Hcoe-coral and 13,025 (50%) Hcoe-symbiont peptide sequences were homologous to NCBI-RefSeq proteins. Among peptides from the Hcoe-specific set, 27,324 (64%) had blastp matches to NCBI-RefSeq proteins, the majority (56%) of which were from metazoans (Fig. 1b, Supplementary Table 6). Comparison of predicted peptides against the OrthoMCL database^23^ revealed that most of the transcriptome sequences cluster with eukaryote protein families (Supplementary Table 7).

Gene ontology analysis revealed that Hcoe-coral transcripts are enriched for typical metazoan cellular processes, such as cell communication, signal transduction, cellular response to stimuli, and regulation of gene expression (Fig. 1d)^24^. On the other hand, Hcoe-symbiont transcripts are enriched for metabolic processes, including carbohydrate and amino acid synthesis, and photosynthesis. Hcoe-specific transcripts are enriched for exocytosis and secretion-related functions.

PFAM domains were identified in 48,834 (54%) of predicted peptides from the *H. coerulea* transcriptome. Global comparisons of PFAM domain composition of various taxa revealed the greatest similiarity of the domain complement of predicted peptides from Hcoe-coral transcripts with that of other cnidarians (Supplementary Figure 1). On the other hand, the PFAM domain composition of peptides from Hcoe-symbiont transcripts was more similar to that of other *Symbiodinium* species. PFAM domains that are most abundant in the Hcoe-coral peptides include those involved in signaling (14-3-3, Ras, Arf, GTP, G-alpha), cytoskeleton and transport (adaptin, microtub_bd, CH), scavenger receptors (SRCR), extracellular matrix (EGF_2), heat shock proteins, and antioxidants (Cpn60_TCP1, HSP90, thioredoxin, redoxin) (Fig. 1e). Abundant PFAM domains in Hcoe-symbiont peptides include antioxidants (glutaredoxin, thioredoxin), methyltransferases, and calcium-binding domains (EF_hand, SPARC_Ca_bdg) (Fig. 1f).

### Divergence time estimation

**Figure 2:**
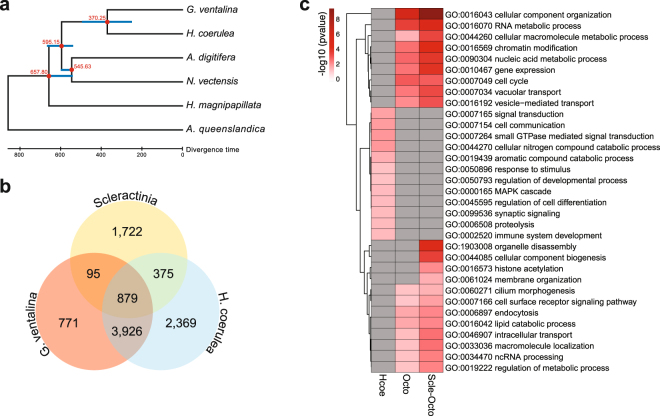
Comparison of the *H. coerulea* gene complement with those of other corals. (**a**) Divergence time estimates for *H. coerulea* and other cnidarians. Phylogenetic relationships among taxa were reconstructed using PhyML with the sponge, *A. queenslandica*, as an outgroup. Red circles at the nodes represent 100% support in PhyML analysis. Divergence was estimated using the Reltime method in MEGA 7. Approximate divergence times of each lineage are shown at the nodes. Blue horizontal bars at each node represent 95% confidence intervals. (**b**) Numbers of orthologous protein groups in *H. coerulea* (Hcoe-coral and Hcoe-specific peptides only)*, G. ventalina*, and scleractinians, as determined by OrthoMCL analysis. (**c**) Gene ontology (GO) analysis for orthologous protein families found in both scleractinians and octocorals (Scle-Octo), octocorals only (Octo), and *H. coerulea* only (Hcoe). Enrichment p-values for selected terms are shown. Only GO terms with a p-value < 0.05 (Fisher’s exact test) were considered significantly enriched.

Phylogenetic analysis was conducted based on alignment of 62,390 amino acid positions from peptides predicted from transcripts originating from the coral host. This estimated the divergence of the blue coral from the sea fan, *G. ventalina*, at about 370 MYA (Carboniferous period) (Fig. 2a). The octocorals diverged from the hexacorals around 595 MYA (Precambrian period). These findings support divergence estimates based on mitochondrial protein-coding genes^25,26^.

### Gene functions in orthologous groups

OrthoMCL analysis was conducted to determine the number of shared and unique orthologous protein families in *H. coerulea, G. ventalina*, and scleractinians. Using OrthoMCL, 10,407 ortholog groups were identified from 69,757 coral proteins. Of these, 879 groups were common to the octocorals, *H. coerula* and *G. ventalina*, and the scleractinians (Fig. 2b). 3,926 gene groups were shared only between the two octocorals. 375 protein families were common only to scleractinian corals and *H. coerulea*. 2,369 gene groups were unique to *H. coerulea*.

Ortholog groups shared by scleractinians and octocorals included gene families associated with cellular component organization, metabolic processes, chromatin modification, gene expression, cell cyle, and transport (Fig. 2c). A similar set of gene ontology terms was enriched among the octocoral-specific ortholog groups. Gene families associated with cell communication, signal transduction, response to stimulus, and development were enriched in ortholog groups unique to *H. coerulea*. In particular, signaling pathways that are regulated by mitogen-activated protein kinases (MAPKs) and small GTPases were overrepresented.

### ITS2 identification and metabolic pathway analysis

The *Symbiodinium* hosted in the tissues of *H. coerulea* from Bolinao, Philippines was found to be most closely related to the thermally tolerant *S. thermophilum* C3-Gulf ITS2 type. Two ITS2 sequences were recovered from *H. coerulea* by DGGE. One sequence was 99.5% identical to the partial *S. thermophilum* ITS2 sequence and the other was 92.6% identical. Both ITS2 sequences contained an 8-bp insertion similar to C3-Gulf ITS2^27^ (Supplementary Figure 2). No other ITS2 types were identified in the coral using DGGE and we did not detect assembled sequences of nuclear rDNA in the transcriptome dataset.

**Figure 3:**
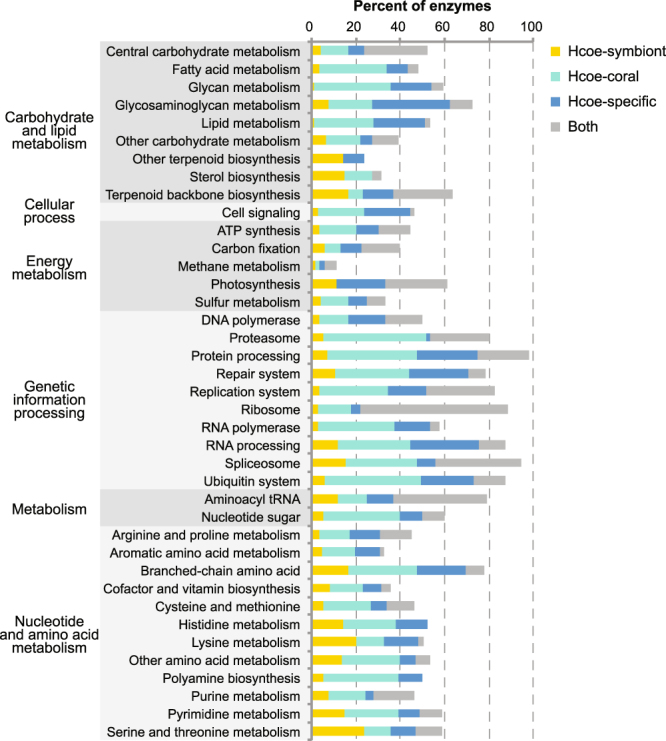
Metabolic complementation analysis for the *H. coerulea* metatranscriptome. Percentages of enzymes in selected KEGG pathways that are represented by transcripts originating from the coral symbiont (Hcoe-symbiont, yellow), transcripts originating from the coral host (Hcoe-coral, light blue), transcripts specific to the *H. coerulea* holobiont (Hcoe-specific, dark blue), and transcripts present in both host and symbiont (Both, grey).

Identification of enzymes within KEGG orthology (KO) pathways that were present in either member of the holobiont or in both revealed biochemical processes that may be performed principally by the host coral or by the symbiont. Examples of modules that are represented mainly in the host are cell signaling and RNA processing (Fig. 3). Enzymes for metabolism of glycans and glycosaminoglycans, which are key extracellular matrix components, are also more highly represented in the host. On the other hand, modules with more enzymes represented only in symbiont transcripts include terpenoid biosynthesis and photosynthesis, as well as pathways for metabolism of amino acids such as lysine, histidine, serine, and threonine.

Key enzymes for amino acid biosynthesis, including diaminopimelate decarboxylase and aspartate kinase for lysine synthesis, glutamine amidotransferase, ATP phosphoribosyltransferase, and histidinol dehydrogenase for histidine synthesis, homoserine kinase and threonine aldolase for serine and threonine synthesis, 5-methyltetrahydrofolate-homocysteine methyltransferase for methionine synthesis, and acetolactate synthase for branched chain amino acid synthesis, were represented in symbiont-derived transcripts but not in host coral-derived transcripts (Supplementary Table 8).

### Biomineralization-related genes

**Figure 4:**
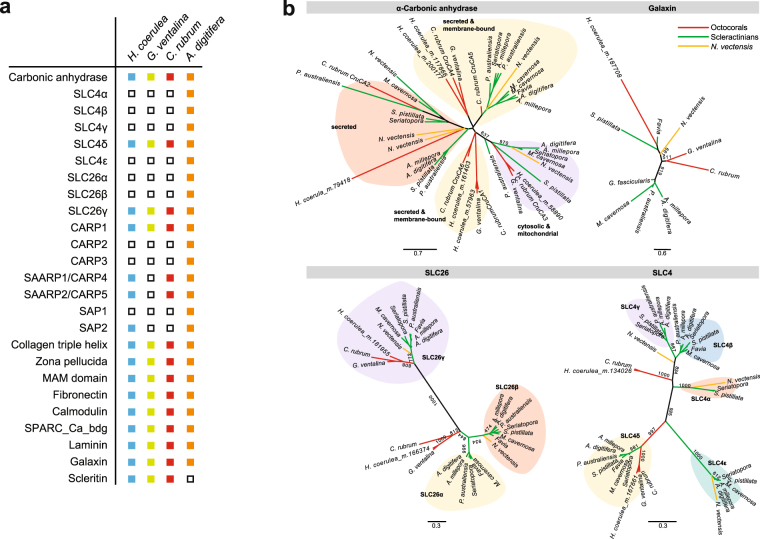
Biomineralization genes in *H. coerulea*. (**a**) Biomineralization-related genes that are represented in the *H. coerulea* transcriptome and those of other anthozoans (present, colored boxes; not detected, white boxes). (**b**) Phylogenetic analyses of biomineralization-related genes present in *H. coerulea*. The unrooted trees were derived from PhyML analysis. Numbers on selected branches represent maximum likelihood bootstrap values.

Coral biomineralization requires sources of inorganic carbon and calcium. Hydration of inorganic carbon to bicarbonate is mediated by carbonic anhydrases. Secreted or membrane-bound, cytosolic, and mitchondrial alpha-carbonic anhydrase (α-CA) types are present in the *H. coerulea* transcriptome and in the transcriptomes of other octocorals, *G. ventalina* and *C. rubrum* (Fig. 4a). Phylogenetic analysis revealed that α-CAs from these three octocorals cluster together (Fig. 4b). Representatives of α-CA isoforms, CruCA4 and CruCA6 of *C. rubrum*, were identified in the *H. coerulea* transcriptome. CruCA4 is the top gene candidate involved in calcification in *C. rubrum* while CruCA6 is expressed at lower levels in tissues of this soft coral^28^. No homolog of α-CA isoform CruCA5, the most ubiquitous isoform in *C. rubrum*, was identified in the *H. coerulea* transcriptome. Interestingly, four of the six α-CA homologs detected in the *H. coerulea* transcriptome are derived from the Hcoe-specific set of transcripts.

The concentration of bicarbonate anions at the site of calcification is mediated by bicarbonate transporters^29^, such as SLC4 and SLC26. SLC4γ, which is reported to be a key scleractinian-specific gene for calcification^30^, was not detected in *H. coerulea* (Fig. 4b). However, the *H. coerulea* transcriptome included a homolog of SLC4δ, which is present in many cnidarians, as well as SLC26γ, a protein that is highly expressed in the calicoblastic ectoderm of the scleractinian coral, *Stylophora pistillata*^30^. Octocorals possess additional SLC4 and SLC26 homologs that do not cluster closely with scleractinian genes.

Precipitation of calcium carbonate or aragonite crystals is facilitated by organic matrix proteins such as coral acid-rich proteins (CARPs) and skeletal acidic proteins (SAPs). Homologs of CARPs are present in the *H. coerulea* transcriptome (Fig. 4a, Supplementary Figure 3). An acid-rich peptide with low percent identity (40%) to *A. millepora* SAP2 was also identified in the blue coral transcriptome (Fig. 4a). This may be a putative homolog, although SAP proteins were previously reported to be restricted to acroporids^31^. Organization of aragonite crystals into the stable macrostructure that characterizes the coral skeleton is mediated by other organic matrix proteins, such as adhesion proteins (collagen triple helix and zona pellucida domain-containing proteins), lectin-containing proteins (lectin, MAM, and fibronectin-containing proteins), and proteins associated with calcium ion-binding (calmodulin, SPARC_Ca_bdg, EGF, and laminin G domain-containing proteins) all of which were detected in *H. coerulea* (Fig. 4a). In addition, the *H. coerulea* transcriptome was found to contain a homolog of galaxin, a component of the skeletal organic matrix protein first identified in the coral, *Galaxea fascicularis*^32^. The *H. coerulea* galaxin homolog clusters with galaxins from the hard corals, *Favia sp*. and *S. pistillata* (Fig. 4b). Furthermore, a homolog of scleritin, a secreted protein in the sclerite organic matrix of the soft coral, *C. rubrum*, was detected in the *H. coerulea* transcriptome (Fig. 4a).

## Discussion

The blue coral, *H. coerulea*, is unique among octocorals for its ability to produce a massive skeleton of crystalline aragonite. It has a shallow depth range and a higher optimal growth temperature that suggests greater resilience to warming environments than scleractinian corals. These features make this coral an interesting model organism for understanding environmental responses, adaptation to elevated temperature, and biomineralization.

*H. coerulea* has been described as a living fossil because its skeletal features remain unchanged from the oldest representative of the genus, *Heliopora japonica*, of the Lower Cretaceous^33^. Octocorals are thought to have originated from a Precambrian ancestor that diverged 696 MYA, with an approximately 400 MY gap until the appearance of the ancestor of modern octocoral species that emerged around 241 MYA^25^. Our divergence estimation based on transcriptome data shows that the divergence between *H. coerulea* and *G. ventalina* occurred around 370 MYA. This suggests that octocorals may have originated earlier than what was estimated based on mitochondrial protein-coding genes and the fossil record^25^.

The *H. coerulea* metatranscriptome includes transcripts originating from the coral animal or its symbiotic dinoflagellate, *Symbiodinium*. These transcript sets were enriched for signature functions of animals and photosynthetic organisms, respectively. Comparison of orthologous protein families further revealed that, while the blue coral transcriptome includes many functions that are shared with scleractinians and other octocorals, gene families that are unique to *H. coerulea* are enriched for potential roles in signal transduction. This is also reflected in the abundance of protein domains associated with signaling in the blue coral. The expanded repertoire of signaling genes may contribute to its ability to sense and respond to various stimuli in its environment. In addition, the enrichment of transcripts with functions related to cytoskeleton, extracellular matrix, and cell cycle in the blue coral transcriptome suggests a capacity for tissue growth and remodeling that may partly explain the morphological plasticity of this species^34^ and its ability to compete for space on the reef.

The *H. coerulea* transcriptome contains representatives of gene families that are commonly associated with stress responses. This includes an abundance of transcripts with heat shock protein and antioxidant domains that may be important for maintenance of cellular homeostasis. Similar genes are differentially expressed in scleractinians, suggesting a link between expression levels of these genes and bleaching resistance of corals^35–37^. Expression of protective genes in scleractinians is rapid and highly coordinated, indicating a strong transcriptional response to thermal stress in the coral host^38^. Whether this is also the case in *H. coerulea* remains to be determined.

Octocoral species exhibit various degrees of thermotolerance, depending on the presence or absence of symbionts and on the specificity of symbiosis^39,40^. In symbiotic octocorals, the coral hosts are more adapted to higher seawater temperatures than their zooxanthellae^4^. The ability of the holobiont to withstand environmental stressors may be related to the degree of interdependency between the coral host and the symbionts^20,21^. Many coral species are capable of hosting multiple types of *Symbiodinium* that vary in their susceptibility to bleaching. Changes in the endosymbiont community composition to include more thermotolerant clades may increase resistance to future bleaching^41^. *H. coerulea* from the Andaman Sea, in the northeastern Indian Ocean, hosts thermally tolerant symbionts of ITS2 type D2-4-5^42^, while the same species in Guam hosts generalist symbionts of type C3^43^. The present study revealed that *H. coerulea* from Bolinao, Pangasinan, host symbionts of the C3-Gulf ITS2 type, a thermotolerant lineage previously detected in corals from the Persian Gulf, where temperatures can reach high seasonal maxima. This study provides the first evidence for this ITS2 variant in a coral outside of the Persian/Arabian Gulf region. This finding suggests that the high optimal growth temperature of *H. coerulea* may be supported by the presence of thermally-tolerant symbiont types in its tissues.

Co-evolution of host and symbionts results in genomic changes in both symbiotic partners. In the coral holobiont, this is evidenced by metabolic complementation between the coral and *Symbiodinium*, with about 80% of the daily photosynthate transferred from the symbiont to the scleractinian coral host. In octocorals, the degree of metabolic exchange with symbionts varies depending on colony morphology, depth range, and symbiont specificity^40^. Transcriptome analysis revealed that the blue coral lacks enzymes for key steps in amino acid metabolic pathways and probably relies on its symbionts for amino acids that it is unable to produce. This is similar to what has been shown in *A. digitifera*, *P. australiensis*, and *G. fascicularis*^20,21,44^. It should be noted, however, that the inability to detect representatives for certain enzymes involved in metabolic pathways may not necessarily indicate their absence from the coral or symbiont genome. It may simply be that they were not captured in the tissues used for transcriptome sequencing. In addition to acquiring photosynthates from their symbionts, corals can augment their nutrient supply through heterotrophic feeding^45^. Octocorals, in particular, are known to exhibit mixed phototrophic and heterotrophic nutrition to sustain their metabolic needs^40,46,47^. Heterotrophy may reduce host dependency on endosymbionts thereby allowing them to allocate energy toward repair and recovery from stress. However, it remains to be determined whether the blue coral exhibits extended heterotrophic compensation as an adaptive strategy to survive adverse conditions, as has been reported in other corals^40,48^.

Evolution of calcification has been linked to the presence of bicarbonate transport proteins, expansion of the secreted and membrane-associated carbonic anhydrase repertoire, and recruitment of extracellular matrix-derived genes to regulate mineralization^30,49^. In scleractinians, precipitation of aragonite, which occurs under the calicoblastic ectodermal layer, is mediated by the concentration of inorganic carbon and calcium ions and proteins that catalyze the nucleation process and crystallization^29,50,51^. The blue coral skeleton, like that of scleractinians, is composed of fibrous aragonite instead of the typical calcitic sclerite found in soft corals. An *in situ* microRaman spectral mapping study revealed the regular cyclic secretion of organic and mineral components in biomineralization zones of the blue coral^52^. This suggests that *H. coerulea* skeleton fiber growth is induced by the organic matrix. Manual curation of the *H. coerulea* transcriptome revealed coral biomineralization-related genes, including carbonic anhydrases, bicarbonate transporters, calcium-binding proteins, and skeletal organic matrix proteins (SOMPs). Representative SOMPs involved in various stages of skeleton formation in *A. digitifera*^31^ were identified in the *H. coerulea* transcriptome, along with other extracellular matrix proteins and proteins involved in secretion and exocytosis. The *H. coerulea* biomineralization gene repertoire shares similarities with both scleractinians and other octocorallians, although some key scleractinian biomineralization genes were not detected in the transcriptome. Genome sequencing of *H. coerulea*, as well as other octocorals, would provide a definitive answer as to whether the genes are indeed absent in these taxa. The presence of homologs of biomineralization enzymes, transporters, and organic matrix proteins, such as galaxin, that are typically found in scleractinian corals may underlie the ability of the blue coral to form massive aragonite skeletons. However, differences in the activities and biochemical characteristics of these proteins may result in the production of skeletons with different properties compared to those of scleractinian corals. Further studies are needed to understand the roles of these genes in *H. coerulea* biomineralization.

The *H. coerulea* gene complement shares many similarities with both scleractinians and other octocorals. The presence of genes that function within known pathways or networks highlight genetic mechanisms that potentially govern interactions of the blue coral with its environment. Additional experiments need to be conducted to determine how this gene complement responds to changing ocean conditions. The transcriptome resource presented in this study thus serves as a springboard for further investigations into the physiology of the blue coral and its environmental responses. These studies will contribute to a deeper understanding of the phylogeny, character evolution, and key innovations within the Octocorallia.

## Methods

### Coral collection

Blue coral colonies were collected at a depth of 10–15 meters by scuba diving in Lucero, Bolinao, Pangasinan, Philippines (N 16°2441 E 119°5420) in January 2015 (Supplementary Table 1). *H. coerulea* exists in several forms, but only the digitate form was used in this study. Collections were conducted with permission from the Philippines Department of Agriculture Bureau of Fisheries and Aquatic Resources (DA-BFAR GP-0097-15). Colonies were temporarily placed in tanks with flowing 10 um-filtered seawater. To avoid excessive mucus production during fragment collection, care was taken to minimize air exposure of the colonies. Coral fragments were immediately flash-frozen in liquid nitrogen after collection, prior to transport and subsequent molecular analyses.

### RNA extraction and mRNA sequencing

Total RNA from coral fragments originating from three biologically independent coral colonies was extracted using Trizol (Invitrogen) following the manufacturer’s protocol. Coral fragments (~2.5 cm long) from each coral colony were manually homogenized using a mortar and pestle. Contaminating genomic DNA in the RNA extracts was removed using a Turbo DNA-free kit (Life Technologies) followed by ethanol precipitation. Nucleic acid concentrations were quantified using a Qubit 3.0 Fluorometer (Life Technologies). RNA integrity was assessed by electrophoresis on native agarose gels with denaturing loading dye and using an Agilent Bioanalyzer 2100 (Supplementary Table 2). RNA samples were sent to the Beijing Genomic Institute (BGI), Hong Kong, for preparation of barcoded libraries using the Illumina TruSeq RNA Sample Prep Kit V2 protocol. mRNA was polyA-selected from total RNA and fragmented followed by first- and second-strand synthesis to produce products ready for library construction. mRNA-enriched libraries were sequenced on the Illumina HiSeq 2000 platform generating 100 bp paired-end reads with all cDNA libraries pooled in a single lane.

### Transcriptome assembly and post-assembly filtering

To detect a greater number of genes expressed by the coral holobiont, reads from three biologically independent coral colonies were pooled and assembled. Raw sequence reads were filtered to remove adapter sequences and low-quality reads. Trimmomatic 0.36 was used to trim the first 10 bases of the reads^53^. Reads were scanned using a 4-base sliding window, deleting bases when the average quality dropped below 20. Bases with quality scores below 30 at leading and trailing ends were removed. Only reads that passed the quality filters and were longer than 30 bases were retained for further analysis. *De novo* assembly was carried out using Trinity software V.2.1.1^54^. Transcripts shorter than 300 bp were removed as these were potentially misassembled sequences or did not encode proteins. Coding regions within the transcripts were identified using TransDecoder V.2.0.1. Only the longest ORF (>100 amino acids) from each transcript was included in the *H. coeruela* transcriptome reference peptide set. CDHIT was then implemented using default parameters to reduce redundancy among proteins (95% similarity and word size of 10)^55^.

### Transcriptome annotation

The *H. coerulea* transcriptome was aligned with proteins in the UniProt-SwissProt and NCBI-RefSeq databases using blastp with an e-value cutoff of 1 × 10^−5^. The top hit from the NCBI-RefSeq blastp alignment was used as input for Blast2GO to retrieve gene ontology terms. Protein domains were identified by mapping predicted peptides against the PFAM 28.0 database using HMMER v3.1b1.

### Classification of coral and symbiont transcripts

To identify transcripts originating from the coral host or the symbiont, the *H. coerulea* reference assembly was aligned by blastn to customized databases. The cnidarian database included the genomes of *A. digitifera* (scleractinian)^21^, *Aiptasia* (sea anemone)^56^, *Hydra magnipapillata*^57^, *Discosoma sp*.^58^, and *Amplexidiscus fenestrafer*^58^ while the *Symbiodinium* database consisted of the *S. minutum*^59^*, S. kawagutii*^60^, and *S. microadriaticum*^22^ genomes, as well as transcriptomes of *Symbiodinium* cultures (clades A1, A2, B2, C and D) (Supplementary Table 3)^61,62^. Transcripts that aligned only to cnidarian polynucleotides were classified as cnidarian sequences (Hcoe-coral) while those that aligned only to *Symbiodinium* polynucleotides were classified as symbiont sequences (Hcoe-symbiont). Sequences that had hits to both databases were further segregated using the psytrans script (https://github.com/sylvainforet/psytrans). Unclassified sequences that did not align to sequences in either database, were regarded as Hcoe-specific. Different e-value cutoffs (1 × 10^−4,^, 1 × 10^−5^, 1 × 10^−8^, 1 × 10^−10^) were examined and the cutoff that resulted in a minimum number of unclassified transcripts was chosen (Supplementary Table 4).

GO enrichment analysis for binned transcripts was performed using the topGO package in R. Only GO terms with a p-value < 0.05 were considered significantly enriched. Enriched PFAM domains in Hcoe-coral and Hcoe-symbiont sequences were also identified. Log-transformed, normalized PFAM domain counts were visualized as heatmaps using pheatmap in R.

### Divergence time estimation

Peptides from six taxa (*H. coerulea, G. ventalina, A. digitifera, H. magnipapillata, N. vectensis*, and *A. queenslandica*) were assigned into ortholog groups (OGs) using a hidden Markov model-based search with HaMStR. Only Hcoe-coral peptides were used for this analysis and only OGs shared by all six taxa were selected. Each OG was aligned using MAFFT v7.220 (parameters:–localpair–maxiterate 1000), and then alignments were trimmed using TrimAl v1.2 with a gap threshold of 0.9, a similarity threshold of 0.001, and a window size of 6. Trimmed alignments shorter than 30 amino acids were discarded. Ortholog alignments were concatenated into a supermatrix using FASconCAT-G. There were a total of 60,627 positions in the final dataset. The best substitution model was determined using ProtTest (v3.4). Maximum-likelihood analysis was implemented on PhyML 3.062 with 1,000 bootstrap replicates. Reltime ML using the JTT matrix-based model was run on MEGA 7 to calculate the divergence time with *A. queenslandica* (Porifera) as the outgroup. The reference divergence times of *H. magnipapillata, N. vectensis, G. ventalina*, and *A. digitifera* were obtained from TimeTree website.

### OrthoMCL analysis and orthologous group enrichment analysis

Orthologous gene groups were identified using OrthoMCL with default settings (e-value cutoff 1 × 10^−5^, protein identity 50%, and MCL inflation of 1.5). The *G. ventalina* and scleractinian data sets contained 31,334 and 16,249 peptides, respectively. The *H. coerulea* dataset included 64,732 peptides from the Hcoe-coral and Hcoe-specific clusters. Only ortholog groups with three or more members were considered. The scleractinian ortholog data set, which includes sequences from 20 hard coral species, was downloaded from comparative.reefgenomics.org^63^. Enrichment analysis for species and lineage-specific gene groups was performed using the topGO package in R. Only GO terms with a p-value < 0.05 were considered significantly enriched.

### Denaturing Gradient Gel Electrophoresis

Total DNA from the coral holobiont was extracted using a modified CTAB method. Briefly, small coral fragments from three biologically independent coral colonies were homogenized with a mortar and pestle in CTAB extraction buffer (100 mM TrisCl, pH 8.0, 20 mM EDTA, 2% CTAB, 1.4 M NaCl, 2.5 mg/mL lysozyme) and incubated at 37 °C for 40 mins. After addition of 0.2% β-mercaptoethanol and 0.1 mg/mL proteinase K, samples were incubated at 60 °C for 1 hr, followed by chloroform fractionation and isopropanol precipitation^64^. The DNA pellet was washed with 70% ethanol and dried at room temperature. DNA was dissolved in 1× TE buffer and stored at −20 °C. PCR amplification of the internal transcribed spacer 2 (ITS2) region was performed using the primers ITSinfor2 and ITS2CLAMP^65^. Fragments were resolved on a 30–60% linear gradient of urea and formamide with 8% acrylamide^66^. Gels were run in 1× TAE at 60 V for 16 hours at 60 °C in a DGGE apparatus (C.B.S. Scientific). After electrophoresis, the gel was stained with SYBR Gold nucleic acid stain in 1× TAE buffer, rinsed, and photographed. Distinct bands were excised from the gel and placed in 30 uL of nuclease-free water overnight to diffuse the DNA. Eluted DNA was re-amplified using ITSinfor2 and ITSrev^65^. Reaction products were sent to First Base, Malaysia for direct sequencing.

### Metabolic complementation analysis

Sequences in the Hcoe-coral, Hcoe-symbiont, and Hcoe-specific transcript sets were compared to enzyme sequences in the curated Kyoto Encyclopedia of Genes and Genomes (KEGG) to identify homologs of enzymes in different metabolic pathways. For each KEGG pathway, the percentage of enzymes represented by coral or symbiont transcripts was calculated using a custom Perl script.

### Phylogenetic analyses

Protein sequences were aligned using Clustal Omega^67^ and trimmed with Gblocks^68^. The best substitution model for each alignment was determined using ProtTest (v3.4). Maximum-likelihood analysis was implemented on PhyML 3.062 with 1,000 bootstrap replicates. Genes used for phylogenetic comparisons were identifed through reciprocal blastp.

### Accession numbers

Raw sequence reads were deposited in the NCBI Short Read Archive database (PRJNA397991). The Transcriptome Shotgun Assembly project has been deposited at DDBJ/ENA/GenBank under the accession GFVH00000000. The version described in this paper is the first version, GFVH00000000.

## Electronic supplementary material

Supplementary information
