# Supplementary material for: Transcriptome analysis of the reef-building octocoral, Heliopora coerulea
Source: Sci Rep. 2018 May 30;8:8397. doi: 10.1038/s41598-018-26718-5 (PMC5976621; doi:10.1038/s41598-018-26718-5)
Supplement: Supplementary file 1 — Supplementary information [file 41598_2018_26718_MOESM1_ESM.pdf]

Supplementary Information

Transcriptome analysis of the reef-building octocoral, *Heliopora coerulea*

Christine Guzman[1,2], Chuya Shinzato[3], Tsai-Ming Lu[2], and Cecilia Conaco[1*]

[1]Marine Science Institute, College of Science, University of the Philippines, Diliman, Quezon City 1101, Philippines

[2]Okinawa Institute of Science and Technology Graduate University, 1919-1 Tancha, Onna-son, Kunigami-gun, Okinawa 904-0495, Japan

[3]Department of Marine Bioscience, Atmosphere and Ocean Research Institute, The University of Tokyo, Kashiwa-shi, Chiba 277-8564, Japan

*Corresponding author: Cecilia Conaco, Marine Science Institute, University of the Philippines, Diliman, Quezon City 1101, Philippines, 6324332991, cconaco@msi.upd.edu.ph

List of Supplementary Information

Supplementary Figures

Figure 1 Comparison of PFAM domain composition in predicted peptides from the indicated organisms.

Figure 2 Alignment of ITS2 type C sequences.

Figure 3 Phylogenetic analyses of coral acid-rich proteins (CARPs).

Supplementary Tables

Table 1 Details of coral collection and RNA quality.

Table 2 *Heliopora coerulea* transcriptome assembly statistics.

Table 3 Sources of cnidarian and *Symbiodinium* sequence databases used in the blastn analysis.

Table 4 Classification of *H. coerulea* transcripts as originating from either the coral host or symbiont.

Table 5 Number of *H. coerulea* transcripts annotated using public databases.

Table 6 Taxonomic assignment of the top blast hit for transcripts with no similarity to cnidarian and symbiont databases (Hcoe-specific set).

Table 7 Comparison of *H. coerulea* predicted peptides against proteins in the OrthoMCL database.

Table 8 Amino acid biosynthesis enzymes represented in symbiont-derived transcripts only.

Supplementary Note

Supplementary Figures

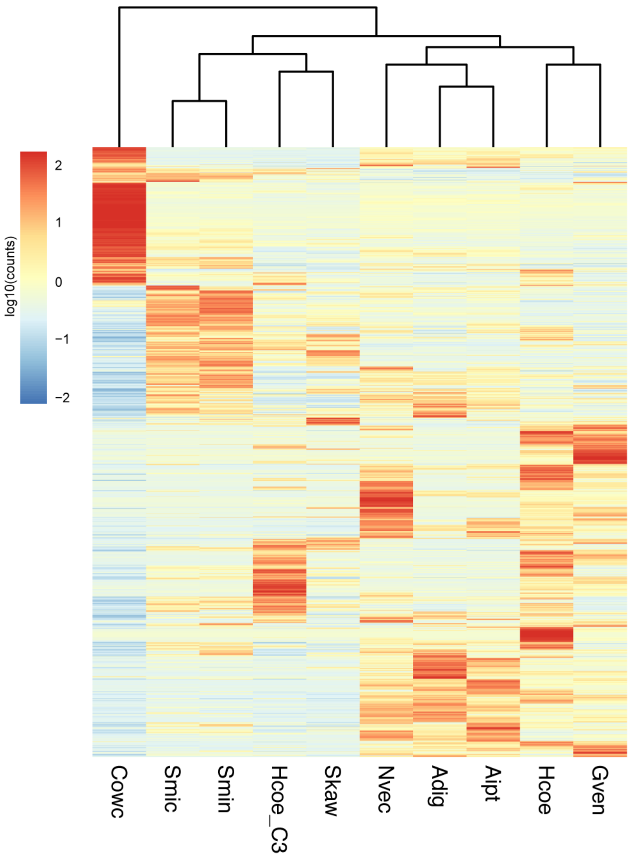

Supplementary Figure 1. Comparison of PFAM domain composition in predicted peptides from the indicated organisms. Species names are abbreviated as follows: *H. coerulea* coral host (Hcoe), *G. ventalina (Gven), N. vectensis (Nvec), A. digitifera (Adig), A. pallida (Apal), H. coerulea* symbionts *(Hcoe-s), C. owczarzaki (Cowc), S. kawagutii (Skaw), S. microadriaticum (Smic),* and *S. minutum (Smin).*

```
                                                          20                    40                    60
                                                           |                     |                     |
  Hcoe MF682429  TTGCCCCAAC TTTGCAAGCA GGATGTGTTT CTGCCTTGCG TTCTTATGAG CTATTGCCCT 60
  Hcoe MF682430  TTGCCCCAAC TTTGCAAGCA GGATGTGTTT CTGCCTTGCG TTCTTATGAG CTATTGCCCT 60
KP234524_C3Gulf  TTGCCCCAAC TTTGCAAGCA GGATGTGTTT CTGCCTTGCG TTCTTATGAG CTATTGCCCT 60
Hcoe_C3 FM877427 TTGCCCCAAC TTTGCAAGCA GGATGTGTTT CTGCCTTGCG TTCTTATGAG CTATTGCCCT 60
            C15  TTGCCCCAAC TTTGCAAGCA GGATGTGTTT CTGCCTTGCG TTCTTATGAG CTATTGCCTT 60
             C3  TTGCCCCAAC TTTGCAAGCA GGATGTGTTT CTGCCTTGCG TTCTTATGAG CTATTGCCCT 60
           Cspc  TTGCCCCAAC TTTGCAAGCA GGATGTGTTT CTGCCTTGCG TTCTTATGAG CTATTGCCCT 60
           Cspa  TTGCCCCAAC TTTGCAAGCA GGATGTGTTT CTGCCTTGCG TTCTTATGAG CTATTGCCCT 60
            C3w  TTGCCCCAAC TTTGCAAGCA GGATGTGTTT CTGCCTTGCG TTCTTATGAG CTATTGCCCT 60
             C1  TTGCCCCAAC TTTGCAAGCA GGATGTGTTT CTGCCTTGCG TTCTTATGAG CTATTGCCCT 60
          C3.10  TTGCCCCAAC TTTGCAAGCA GGATGTGTTT CTGCCTTGCG TTCTTATGAG CTATTGCCCT 60
            C3e  TTGCCCCAAC TTTGCAAGCA GGATGTGTTT CTGCCTTGCG TTCTTATGAG CTATTGCCCT 60
                 ********** ********** ********** ********** ********** ******** *
                                                          80                   100                   120
                                                           |                     |                     |
  Hcoe MF682429  CTGAGCCAAT GGCTTGTTAA TTGCTTGGTT CTTGCAAAAT GCTTTGCGCG CTGTTATTCA 120
  Hcoe MF682430  CTGAGCCAAT GGCTTGTTAA TTGCTTGGTT CTTGCAAAAT GCTTTGCGCG CTGTTATTCA 120
KP234524_C3Gulf  CTGAGCCAAT GGCTTGTTAA TTGCTTGGTT CTTGCAAAAT GCTTTGCGCG CTGTTATTCA 120
Hcoe_C3 FM877427 CTGAGCCAAT GGCTTGTTAA TTGCTTGGTT CTTGCAAAAT GCTTTGCGCG CTGTTATTCA 120
            C15  CTGCGCCAAT GGCTTGTTAA TTGCTTGGTT CTTGCAAAAT GCTTTGCGCG CTGTTATTCA 120
             C3  CTGAGCCAAT GGCTTGTTAA TTGCTTGGTT CTTGCAAAAT GCTTTGCGCG CTGTTATTCA 120
           Cspc  CTGAGCCAAT GGCTTGTTAA TTGCTTGGTT CTTGCAAAAT GCTTTGCGCG CTGTTATTCA 120
           Cspa  CTGAGCCAAT GGCTTGTTAA TTGCTTGGTT CTTGCAAAAT GCTTTGCGCG CTGTTATTCA 120
            C3w  CTGAGCCAAT GGCTTGTTAA TTGCTTGGTT CTTGCAAAAT GCTTTGCGCG CTGTTATTCA 120
             C1  CTGAGCCAAT GGCTTGTTAA TTGCTTGGTT CTTGCAAAAT GCTTTGCGCG CTGTTATTCA 120
          C3.10  CTGAGCCAAT GGCTTGTTAA TTGCTTGGTT CTTGCAAAAT GCTTTGCGCG CTGTTATTCA 120
            C3e  CTGAGCCAAT GGCTTGTTAA TTGCTTGGTT CTTGCAAAAT GCTTTGCGCG CTGTTATTCA 120
                 *** ****** ********** ********** ********** ********** **********
                                                         140                   160                   180
                                                           |                     |                     |
  Hcoe MF682429  AGTTTCTACC TTCGTGGTTT TACTTGAGTG ACGCTGCTCA TCTTCGCATG CTTGGGAATG 180
  Hcoe MF682430  AGTTTCTACC TTCGTGGTTT TACTTGAGTG ACGCTGCGGC GCTGCTCATG CTTGCAACCG 180
KP234524_C3Gulf  AGTTTCTACC TTCGTGGTTT TACTTGAGTG ACGCTGCGAC GCTGCTCATG CTTGCAACCG 180
Hcoe_C3 FM877427 AGTTTCTACC TTCGTGGTTT TACTTGAGTG AC-------- GCTGCTCATG CTTGCAACCG 172
            C15  AGTTTCTACC TTCGCGGTTT TACTTGAGTG AC-------- GCTGCTCATG CTTGCAACCG 172
             C3  AGTTTCTACC TTCGTGGTTT TACTTGAGTG AC-------- GCTGCTCATG CTTGCAACCG 172
           Cspc  AGTTTCTACC TTCGTGGTTT TACTTGAGTG AC-------- GCTGCTCATG CTTGCAACCG 172
           Cspa  AGTTTCTACC TTCGTGGTTT TACTTGAGTG AC-------- GCTGCTCATG CTTGCAACTG 172
            C3w  AGTTTCTACC TTCGTGGTTT TACTTGAGTG AC-------- GCTGCTCATG CTTGCAACCG 172
             C1  GGTTTCTACC TTCGTGGTTT TACTTGAGTG AC-------- GCTGCTCATG CTTGCAACCG 172
          C3.10  AGTTTCTACC TTCGTGGTTT TACTTGAGTG AC-------- GCTGCTCATG CTTGCAACTG 172
            C3e  AGTTTCTACC TTCGTGGTTT TACTTGAGTG AC-------- GCTGCTCATG CTTGCAACCG 172
                  ********* **** ***** ********** **          ** * **** ****  *  *
                                                         200
                                                           |
  Hcoe MF682429  CTGGGGAGTG CGTGTATGCC TC 202
  Hcoe MF682430  CTGGGATGCA GGTGCATGCC TC 202
KP234524_C3Gulf  CTGGGATGCA GGTGCATGCC TC 202
Hcoe_C3 FM877427 CTGGGATGCA GGTGCATGCC TC 194
            C15  CTGGGATGCA GGTGCATGCC TC 194
             C3  CTGGGATGCA GGTGCATGCC TC 194
           Cspc  CTGGGATGCA GGTGCATGCC TC 194
           Cspa  CTGGGATGCA GGTGCATGCC TC 194
            C3w  CTGGGATGCA GGTGCATGCC TC 194
             C1  CTGGGATGCA GGTGCATGCC TC 194
          C3.10  CTGGGATGCA GGTGCATGCC TC 194
            C3e  CTGGGATGCA GGTGCATGCC TC 194
                 *****  *    *** ***** **
```

Supplementary Figure 2. Alignment of ITS2 type C sequences. The C3 type symbionts identified in *H. coerulea* (NCBI accessions MF682429 and MF682430) and the C3-Gulf type *Symbiodinium thermophilum* from corals in the Arabian/Persian Gulf (NCBI accession KP234524.1) have an 8bp insertion (red box). *H. coerulea* from Guam hosts C3 type symbionts (NCBI accession FM877427.1). Other ITS2 sequences were obtained from the GeoSymbio website.

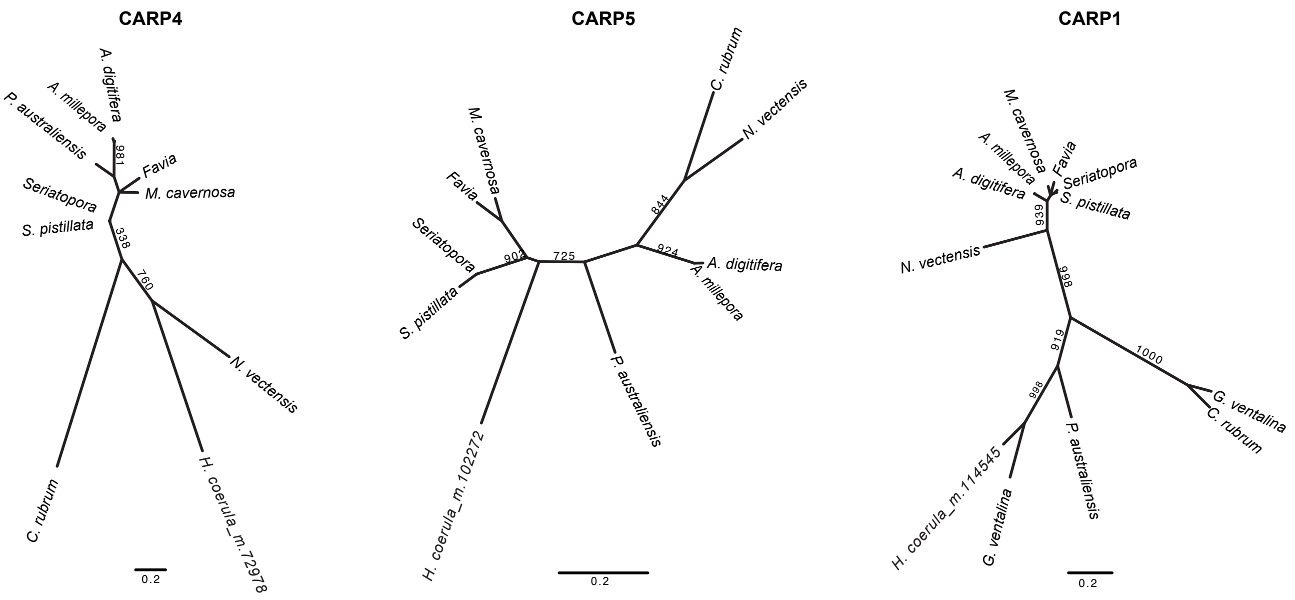

Supplementary Figure 3. Phylogenetic analyses of coral acid-rich proteins (CARPs) present in *H. coerulea*. The unrooted trees were derived from PhyML analysis. Numbers on selected branches represent maximum likelihood bootstrap values.

Supplementary Tables

Supplementary Table 1. Details of coral collection and RNA quality.

Species name	Colony form	Colony #	RIN	28S/18S	Date of collection	Location
Heliopora coerulea	digitate	1	9.0	1.9	January 2015	Bolinao, Pangasinan, Philippines
Heliopora coerulea	digitate	2	8.4	1.3	January 2015	Bolinao, Pangasinan, Philippines
Heliopora coerulea	digitate	3	9.0	1.8	January 2015	Bolinao, Pangasinan, Philippines

Supplementary Table 2. *Heliopora coerulea* transcriptome assembly statistics.

No. of libraries used	3 libraries (1 library per colony)
Reads used	82,760,176
After Trinity assembly	231,515 transcripts (N50=1,106bp)
After TransDecoder	164,516 peptides
Unique peptides (1peptide/transcript)	155,859 peptides
After CDHIT clustering	90,817 peptides

Supplementary Table 3. Sources of cnidarian and *Symbiodinium* sequence databases used in the blastn analysis for assignment of transcriptome sequences to coral holobiont compartments.

	Dataset type	Dataset NCBI accession	Reference
Cnidarian database			
Acropora digitifera	genome	PRJDA67425	Shinzato et al 2011
Hydra magnipapillata	genome	PRJNA12876	Chapman et al. 2010
Aiptasia	genome	PRJNA261862	Baumgarten et al. 2015
Discosoma	genome	PRJNA354492	Wang et al. 2017
Amplexidiscus fenestrafer	genome	PRJNA354436	Wang et al. 2017
Symbiodinium database			
Symbiodinium microadriaticum (clade A)	genome	PRJNA292355	Aranda et al. 2016
Symbiodinium minutum (clade B)	genome	PRJDB732	Shoguchi et al. 2013
Symbiodinium kawagutii (clade F)	genome	PRJNA242224	Lin et al. 2015
Clade A1 (culture)	transcriptome	PRJNA206215	Baumgarten et al. 2013
Clade A2 (culture)	transcriptome	PRJNA237994	Rosic et al. 2015
Clade B2 (culture)	transcriptome	PRJNA260330	Rosic et al. 2015
Clade C (culture)	transcriptome	PRJNA260340	Rosic et al. 2015
Clade D (culture)	transcriptome	PRJNA260333	Rosic et al. 2015

Supplementary Table 4. Classification of *H. coerulea* transcripts as originating from either the coral host or symbiont.

	**Blastn evalue cutoff**			
	**$1x10^{-4}$**	**$1x10^{-5}$**	**$1x10^{-8}$**	**$1x10^{-10}$**
With hits to cnidarian database only	13,396	17,529	16,487	14,338
With hits to *Symbiodinium* database only	23,967	23,739	23,135	23,005
With hits to both databases	7,961	6,991	5,422	4,489
With hits to cnidarian database, using psytrans script	5,334	4,645	3,522	2,935
With hits to Symbiodinium database, using psytrans script	2,627	2,346	1,920	1,554
Transcripts originating from coral (Hcoe-coral)	18,730	22,174	20,009	17,273
Transcripts originating from symbiont (Hcoe-symbiont)	26,594	26,085	25,055	24,559
Unclassified transcripts (Hcoe-specific)	45,493	42,558	45,753	48,985

Supplementary Table 5. Number of *H. coerulea* transcripts annotated using public databases.

Database	Hcoe-coral	Hcoe-symbiont	Hcoe-specific	Total
SwissProt	19,790	9,923	20,083	49,796 (55%)
NCBI-RefSeq	21,573	13,025	27,324	61,922 (68%)
PFAM	18,370	10,856	19,608	48,834 (54%)
KEGG	4,608	2,000	3,757	10,366 (11%)
No annotation				26,207 (32%)

Supplementary Table 6. Taxonomic assignment of the top blastx hit for transcripts with no similarity to cnidarian and symbiont databases (Hcoe-specific set).

Taxonomic assignment	Number of transcripts
Archaea	27
Bacteria	902
Fungi	118
Metazoa	23,706
Other Eukaryotes	497
Viridiplantae	1,898
Viruses	71
Unknown/No annotations	15,339

Supplementary Table 7. Comparison of *H. coerulea* predicted peptides against proteins in the OrthoMCL database (October 2016 version). The number of orthologous protein families common between *H. coerulea* and organisms in other domains are shown.

	Number of protein families
Number of peptides with homology to OrthoMCL groups	17,845
Total number of groups	5,693
Eukarya only	4,337
Bacteria only	0
Archaea only	0
Eukarya + Bacteria	537
Eukarya + Archaea	221
Bacteria + Eukarya	0
Eukarya + Bacteria + Archaea	598

Supplementary Table 8. Amino acid biosynthesis enzymes represented in symbiont-derived transcripts only. Enzyme names are based on KEGG annotation of *H. coerulea* transcripts.

Amino acid synthesis pathway	Enzyme name	Transcript and peptide ID
Lysine	diaminopimelate decarboxylase	TRINITY_DN211972_c0_g1_i1|m.207725, TRINITY_DN260678_c0_g1_i1|m.226325, TRINITY_DN82573_c0_g1_i1|m.34176
	aspartate kinase	TRINITY_DN223075_c0_g1_i1|m.211778
Glysine, Threonine, Serine	bifunctional aspartokinase / homoserine dehydrogenase 1	TRINITY_DN199157_c0_g1_i1|m.201768
	homoserine kinase	TRINITY_DN263580_c0_g1_i1|m.227676
	threonine aldolase	TRINITY_DN139070_c1_g1_i1|m.69474
Cysteine and Methionine	5-methyltetrahydrofolate--homocysteine methyltransferase	TRINITY_DN189853_c0_g1_i1|m.196470, TRINITY_DN192117_c0_g1_i1|m.197794, TRINITY_DN189853_c0_g1_i1|m.196470
	cystathione beta-lyase	TRINITY_DN187740_c0_g1_i1|m.195142
Valine, Leucine and Isoleucine	acetolactate synthase	TRINITY_DN108227_c0_g1_i1|m.48927
	branched-chain amino acid aminotransferase	TRINITY_DN282645_c0_g1_i1|m.235123, TRINITY_DN126983_c0_g1_i1|m.59267
	2-isopropylmalate synthase	TRINITY_DN292157_c0_g1_i1|m.237600, TRINITY_DN6321_c0_g1_i1|m.2183, TRINITY_DN101103_c0_g1_i1|m.45246, TRINITY_DN101103_c1_g1_i1|m.45248
	ketol-acid reductoisomerase	TRINITY_DN141310_c0_g1_i1|m.71383, TRINITY_DN41157_c0_g1_i1|m.15510
Phenylalanine, Tyrosine and Tryptophan	anthranilate phosphoribosyltransferase	TRINITY_DN250856_c0_g1_i1|m.221110
	3-phosphoshikimate 1-carboxyvinyltransferase	TRINITY_DN29042_c0_g1_i1|m.11083
	3-dehydroquinate synthase	TRINITY_DN159188_c0_g1_i1|m.94972, TRINITY_DN24532_c0_g1_i1|m.10038
	chorismate synthase	TRINITY_DN116675_c0_g1_i1|m.53348

Supplementary Note

Amino acid sequence alignments that were used to generate phylogenetic trees.

Alpha-Carbonic anhydrases

```
>ANJ59757.1_alpha_carbonic_anhydrase_2_partial_[Corallium_rubrum]
RFGCTEKTGS EHQIDGVPTP GEIHLVFYK- ---------- -----
>Porites_australiensis_43061
HFGCVNKRGS EHKLNGKQLS GELHMVFGH- GNGY----GN GNGKW
>Hcoerulea_m.57963
HWGGTANTGS EHKFNGQSSS MEVHLVHYNY KGSFTTPPCS ETVTW
>Stylophora_pistillata_15105
HWGSENSRGS EHQVGGRKFP LEIHIVHYNY RGSLTTPGCF ESVQW
>Porites_australiensis_13576
HWGSDDSYGS EHQVLGKKYP LEIHIVHFNY KGSLTTPGCY ESVQW
>Acropora_digitifera_15787
HWGSVDSRGS EHQISGRKYP MEIHIVHFNY KGSLTTPGCY ESVQW
>Acropora_millepora_11476
HWGSVDSRGS EHQISGRKYP MEIHIVHFNY KGSLTTPGCY ESVQW
>Gorgonia_ventalina_58949
HWGDA-HSGS EHKINGKSAS MEVLLVHYNY KGSFTTPPCF ETVSW
>ANJ59756.1_alpha_carbonic_anhydrase_1_partial_[Corallium_rubrum]
HWGNTMSMGS EHKVNGKSSS MEVHLVHYNY QGSFTTPPCL ETVTW
>Stylophora_pistillata_10460
RWGHDNDSGS EHAINDQNYP LEVQLIHWNY QGSLTTPPMS ENVTW
>Gorgonia_ventalina_74222
HWGENNDVGS EHTVDGKPYC GELHLVHYNY LGSLTTPPLS ESVTW
>ANJ59758.1_alpha_carbonic_anhydrase_3_[Corallium_rubrum]
HWGENDDTGS EHTIDGEPYS GELHLVHWNY PGSLTTPPLS ESVTW
>Hcoerulea_m.58890
HWGEDDSSGS EHTVDGNAYA GELHLVHWNY PGSLTTPPLA ESVTW
>Stylophora_pistillata_5598
HFGCDADRGS EHTIDGKSFP AELQLMFYNY MGSLTTPPCY ETVKW
>Nematostella_vectensis_1655
HLGEDDTRGA EHLIDGQRNA ACIHIVNYNY KGSLTTPPCY ESVTW
>Nematostella_vectensis_5378
HWGSKNEQGS EHLIDGKAFA GAIHIVSYNY QGSLTTPGCQ ESVTW
>Montastraea_cavernosa_5903
HFGCDASKGS EHAVDGRVYS GELHLVTYNY KGSLTTPPCY QSVKW
>Nematostella_vectensis_5033
HFGCNDWLGS EHAVDGRRHP GEIQMIFHNY KGSQTAPACH ESVRW
>Montastraea_cavernosa_1262
HWGSSDSKGS EHKVDGKQYA AEIHFVSFNY PGSLTTPSCY ESVLW
>Acropora_millepora_17099
HWGSDNTKGA EHGMDGMFYP AEIHFVSFNY NGSLTTPTCN EAVTW
>Porites_australiensis_6487
HWGLDNNQGS EHTMNEMMYP AEIHFVSYNY PGSLTTPPCS EAVTW
>Porites_australiensis_9041
HWGSNNSQGS EHTLDGKKFS AELHFVSMNY NGSLTTPTCD EAVTW
>Seriatopora_sp_17212
```

```
HWGKTDNVGS EHTMD-MAYP AEIHFVSYNY SGSLTTPTCD ESVTW
>Montastraea_cavernosa_62323
HWGSTNMKGS EHYLDGKQYA AELHFVSYNY SGSLTTPKCQ ESVTW
>Favia_sp_56067
HYGPNNTVGS EHIVDGEHYA AELHFVSYNY SGSLTTPSCT ESVTW
>Acropora_digitifera_14530
HWGANNTVGS EHIVNGKEYA AELHFVSFNY NGSLTTPTCL ESVTW
>Acropora_millepora_11081
HWGANNTVGS EHIVNGKEYA AELHFVSFNY NGSLTTPTCL ESVTW
>Hcoerulea_m.117866
HWGKKNSRGS EHRYSGKVFS AEMHLVHYNY QGSLTTPPCF ETVIW
>ANJ59759.1_alpha_carbonic_anhydrase_4_[Corallium_rubrum]
HWGSDNTQGS EHRFDKERFP AEIHFVHYNY RGSLTTPPCF DHVTW
>Gorgonia_ventalina_30252
HWGSNNQIGS EHQFDGRSFP AELHFVHYNY KGSLTTPPCF ANVIW
>Seriatopora_sp_22437
HFGCENSRGS EHLIDNQSFP AQLHLVFFNY KGSLTTPPCY ESVTW
>Seriatopora_sp_4899
HWGNDDTEGS EHRVDGKMYP AELHFVHWN- ---------- -----
>Nematostella_vectensis_3048
HWGKDEKEGS EHRVDGKMYP SEMHIVHYNY PGSLTTPPLS EIVNW
>Porites_australiensis_31008
HWGASDDHGS EHQVNGKSYA AELHLVHWNY SGSLTTPPCS ESVTW
>Nematostella_vectensis_1606
HVGSSDIQGS EHHIHGVKYP LEMHLVHYNY NGSLTTPPCF ETVKW
>ANJ59760.1_alpha_carbonic_anhydrase_5_[Corallium_rubrum]
HWGSDDSRGS EHKIDGKSYA AELHFVHYNY NGSLTVPNCD ESVTW
>Hcoerulea_m.161403
HWGSDEGKGS EHLINGKHYP AELHLVHYNY QGSLTTPTCD ESVTW
>ANJ59761.1_alpha_carbonic_anhydrase_6_[Corallium_rubrum]
HWGCENGKGS EHLINGLSYP AELHLVHHNY DGSLTTPPCY ESVTW
>Hcoerulea_m.79418
VAQLQLKMPS EHFIDGHAYA AELQVVHRNY YGSSTSPDCH EMNKW
>Montastraea_cavernosa_24886
HWGGNDSEGS EHRVDGKMYP AELHLVHWNY DGSLTTPPLA ECVKW
>Acropora_digitifera_15323
HWGDKDSEGS EHRVNGKMYS AELHFVHWDY DGSLTTPPLL ECVKW
>Acropora_millepora_24165
HWGDKDSEGS EHRVNGKMYS AELHFVHWDY DGSLTTPPLL ECVKW
```

Galaxin

```
>Hcoerulea_m.187708
NCSRIKYIEN VKVKLKIALY GTEKDPYNSD IYECCNDCCS GRIYDKENEL CCAGRVYNK
>Montastraea_cavernosa_17374
CCGSHGYDAA TQLCCSNHPY NTATQLYNTA TQLCCNGCCG TQVYDTRSQI CCDGGTPVP
>Acropora_millepora
CCGRNGYDAN TSLCCGDMGY NRNTHLYSQQ THLCCEGCCG TTSYNPLTEL CCDGIAFFK
>Acropora_digitifera
CCGRNGYDAN TSLCCGDMGY SRNTHLYSQQ THLCCEGCCG TTSYNPLTEL CCDGIAFFK
>Galaxea_fascicularis
CCGPNSYDTN TTLCCGSQGY DGATQLYTQD THLCCEGCCG STSYNPVTEI CCDGHVGTR
>Porites_australiensis_5328
```

CCGSNGYDAN TTLCCGSHGY DGATQLYDQA TQLCCEGCCG PTSFNPSTEI CCDGHASPK
>Stylophora_pistillata_919
CCGRESYDSS HYFCYGKKPY DLVRSLYNSK THFCNKNCCL ESAYDPSIFV CLWGTKIAP
>Corallium_Contig_32924|m.33632
CCDDKNYNPT FDICCGDRLF NPSSKVYNPT FNICCDKCCD EDNYNPNTHV CCDDDILRI
>Favia_sp_47802
CCGRVAYDPS RHVCCGTKLY DYARQLYDPN VQFCCGGCGK SAAYDPATQL CCYEQVVTK
>Nematostella_vectensis
CCGRQTYDNR RYICCGRQTY DNRKYIYNPL TKICCYPCCR YTPYNPLTKI CCYPNILSR
>Gorgonia_ventalina_56329
CCGSINYNPS QYVCCGTQNY DSNTHIYYTG SHVCCGGCCG SSVYRYSSQI CCNNNVLRK

SLC26

>Nematostella_vectensis_8749
GVIQLLMGIL KLGFLVNFIS IPVVSGFTSS AAIIIAISQI KDVLGGLICI IVLGLPPFKA
PIVPLIGFLE SIAIAKAFAR KNRYKVDASQ ELIALGLANV LSSFVSSYPV TGSFSRTAVN
AQSGTPAGGI FTGAIVILAL GVLTPFFKYI PKASLAALII SSVLTMV-EF QIVPRIWRVK
KIDLIPLLVT FFGC-FYEIE YGILAGMGVS LNMSMIQHTD
>Montastraea_cavernosa_10902
GVILLSMGLL RLGFVVNFIS IPVVSGFTTS ASIIIAFSQI KDLLGGLICI LFLGFPPIQA
P--------- ---------- ---------- ---------- ---------- ----------
---------- ---------- ---------- ---------- ---------- ----------
---------- ---------- ---------- ----------
>Acropora_digitifera_8913
-------GFL RLGFVVNFIS IPIVSGFTSS AAVIIAFSQL KDVLGGLICV LVLGLPPIKA
PVVPLIGFLE SIAIAKAFAR KNGYSVDASQ ELIALGIANC LGSFVSSYPV TGSFSRTAVN
AQSGTPAGGI FTGAVVLLAL GVLTPSFKYI PKASLAALIM SSVVTMI-EY HIVPNIWKVR
RLDLVPLAVT FFGC-FYDIE IGILTGIGVA LDLSVVTSID
>Acropora_millepora_7214
GIIQCLLGAL RLGFLVRFIS LPVISGFVSA AAVTIGFGQV KSLLGGFCCI VVLGIPALKA
PVVPLIGFLE SIAIAKAFAR KNGYSVDASQ ELIALGIANC LGSFVSSYPV TGSFSRTAVN
AQSGTPAGGI FTGAVVLLAL GVLTPSFKYI PKASLAALIM SSVVTMI-EY HIVPNIWKVR
RLDLVPLAVT FFGC-FYDIE IGILTGIGVA LDLSVVTSID
>Porites_australiensis_19001
GVVQLFMGTF RLGFLVRFIS LPVISGFVSS AAIIIGFGQV KSLLGGSCCI VILGIPDIRV
PVVPLIGFLE SIAIAKAFAR KNRYSVDASQ ELIALGIANC VSSFVSSYPV TGSFSRTAVN
AQSGTPAGGI FTGAIVLLAL GVLTPSFKYI PKASLAALIM SSVVTMI-EY HILPNIWKVR
RIDLVPLIIT FFGC-FYDIE VGILTGIGVA LDFSVVTSID
>Favia_sp_49105
GIIQLILGLL RLGFVVRFIA LPVISGFVSA AAITIGFGQV KNLLGGSICI VLLGIPHFKA
PVIPLIGFLE SIAIAKAFAR KNRYTVDASQ ELIALGIANC LSSFVSSYPV TGSFSRTAVN
AQSGTPAGGI FTGAVVLLAL GVLTPSFKYI PKASLAALIM SSVVTMI-EY HILPSIWKVR
RIDLVPLVVT FFGC-FYDIE VGILTGIAVA LDFSVVTSID
>Seriatopora_sp_6016
---------- ---------- ---------- ---------- ---------- ---SAEVLLV
PVVPLIGFLE SIAIAKAFAR KNRYTVDASQ ELIALGVANC LSSFVSSYPV TGSFSRTAVN
AQSGTPAGGI FTGAIVLLAL GLLTDSFKYI PKASLAALIM SSVVTMI-EY HIVPNIWKVR
RIDLVPLAIT FFGC-FYDIE VGILAGIAVA LDFSAVTSTD
> Stylophora_pistillata_SLC26gamma
GIILLAMGFL RLGFVVNFIS IPIVSGFTSA ATIIIAFSQL KDLFGGLLCI IILGLPLMQV
PVVPLIGFLE SIAIAKAFAR KNRYTVDASQ ELIALGVANC LSSFVSSYPV TGSFSRTAVN

AQSGTPAGGI FTGAIVLLAL GLLTDSFKYI PKASLAALIM SSVITMI-EY HIVPNIWKVR
RIDLVPLAIT FFGC-FYDIE VGILAGIAVA LDFSLVTSID
>Hcoerulea_m.181955
GLIQLVMGLF SLGFIVRFIP IPVISGFTSS AAIIIGCGQL PGILGGLACV VLLGLPKFGV
PVLPLIGFLE SVAIAKSFAR KNKYKVDPSQ ELIALGVANF IGSFCSAYPV TGSFSRTAVN
SISGTPLGGV FTGGLVLLAV GVLTPFFQYI PKAALGAVIV SSVIHMV-DY RIVKKIWKTN
RTDVIPLFAT FLLC-LYDIA IGIISGIAVA MDCSNMSEVD
>Corallium_rubrum_Contig_12938|m.12629
GIIWLVMGLL SLGFIVRVIP LPVISGFTSS AAIIIACGQL KSILGGLICM VLLGLPAFEV
PVIPMIGFLE DIAIAQVFAR RNHYNVDPSQ ELIALGVANF LGSFVSGYPI TGSFSRTTVN
ALSGTPLGGV FTGCVVLLAL SVLTPFFQYI PDASLASIII VSVLRMV-DL KIVKKIWQTN
RIDIIPLCVT FFVC-FYELD IGIVSGIVVA LDCSAMFECD
>Gorgonia_ventalina_12420
GFFLLFMGFL SLGFIVRLIP VPVISGFTSA ASIIIACEQL PNLLGGVICM AILGLPPFKV
PVVPMIAFLT SIAIVQSFSR KNNYQVNPSQ ELIALGVANV ANSFVSGFPI AGCFSRTTVN
SMTGTPFGGV VTGCVVLLAL SILTPFFQYI PRATLAAVVI VAVLRTV-NI RIIRKIWRIR
KIDVLPLIVT FFAC-LYEIA IGIACGIAMA LDCTSMFECD
>Hcoerulea_m.166374
GIIQTAMGFA RLGFITIFLS DPLISGFTTG AACWVFTSQI KHILAGILCI ALLGLPPISV
PVIAVVAFSV NISLAKLFSQ KHKYPIDANQ ELVAYGVQNI VGCFFSCFVS AGSLGRSLIQ
ENLGSQITSI ISCAVILLVL TALAPLFEPL PNAVLAAIII VAIRRLFKNF LRVKQLWSVN
KIDAFTWVVT WSAVILFGID FGLGIGVVFQ LDCAPISFID
>Gorgonia_ventalina_12168
-----AMGFL RLGFITIFLS DPLISGFTTG AACWVFTSQI KHIIAGILCV LILGLPPLSV
PVISVVAFAV NISLAKLFSQ KHGYPIDANQ ELLAYGVQNI VSGFFSCFVS AASLGRSLIQ
ENLGTQITSV ISCAILLLVL TVLAPLFEPL PNAVLAAIII VAIRRLFKNF VLVGQLWKVN
KIDSVTWVVT WMAVILFGID LGLGIGVFFQ IDCSSFSFID
>Nematostella_vectensis_8308
GIMQILMGLC RLGFVATYLS DPLISGFTTG SAVLVVLSQL KHIFGGVLCL VILGLPPISI
PVISVVIFAT NISLARMFAK KNGQTVDANQ ELLAYGMCNV GGSFFSCFPI CNALARTVVQ
ENLATQLCSI PVICLILLVL LFMAPLFYYL PKAILAAVVI ANLGGLLKQF ARLRQLWCIC
RTDAVTWFVT CFGVILLGVD LGLGLGVITT IDASAFTFID
>Favia_sp_43555
GIIQIVMGAA RLGFVATFMS DPMISGFTTG SAVLVVISQM PHILGGLLCL AILGLPPLSV
PVIAVVIFAT NISVSVMFAK KNGYTVEANQ ELIAYGAGNV AGSFFSCFPI CNALARTAVQ
ENLATQLCDI VVILLIILVL LFMAPLFFHL PKAILAAVVI ANLVGLLKQF ARLRALWKIY
KPDAIVWFLS CFGVILMGVD LGLGIGIICA LDGSSFTFID
>Acropora_digitifera_12
GIFQIIMGAI RLGVLATFMS DPMISGFTTG SAVLVVISQL PHIFGGVLCL LILGLPPISV
PVIAVVIFAT NISVSKMFAK KRGYTIDPNQ ELIAYGVGNV AGSFSSCFAI CNALARTAVQ
ENLATQLCSI VVIVLILLVL LFLAPLFFYL PKAILASVVI ANLIGLLKQF TRLKALWYIY
RPDAVVWFLT CFGVILMGVD LGLGIGVICT LDGSSFTFID
>Porites_australiensis_37404
GIIQIVMGAV HLGVVATYLS DPLISGFTTG SAVLVVISQM KHILGGVLCL AVLGLPPLSV
PVIAVVIFAT NISISKMFAK KHGYLVDPNQ ELIAYGAGNF VGSFFSCFPI CNALARSVVE
DNLATQLCSI VVIVLILLVL LFIAPLFFHL PKAILAAVVI ANLVGLLKQF SRFPVLWRID
KPDAIVWFVS CFGVILMGVD VGLGIGLICA LDGSSFTFID
>Acropora_millepora_6329
GIIQVAMGAA QLGFLATFMS DPMISGFTTG SAVLVVISQV KHILGGVLSL VILGLPPLSL
PVIAVVIFAT NISLSKMFAK KRGYSVDPNQ ELIAYGVGNV AGSFSSCFAI CNALARTAVQ
ENLATQLCSI IVIAMILLVL LFIAPLFFHL PKPILAAVVI ANLVGLLRQF SRLKVLWKIH
KPDAVVWFFT CFGVIAMGVD VGLGIGVMCA LDCSSFTFID
>Montastraea_cavernosa_83900

```
GIIQIVMGAV KLGCVATYLS DPMISGFTTG SAVLVVISQV KLILGGVLSL LVLGLPPVSV
PVIAIVIFAT NISLARMFAK KRGYSVDPNQ ELIAYGAGNL AGSFFSCFPI CNALARTAVQ
ENLATQLCSL VVIALILLVL LFIAQLFFHL PKAILAAVVI ANLIGLLKQF SRLRDLWRIY
KPDAVVWFCS CFGVILLGVD VGLGVGVISA LDCSSFTFID
>Seriatopora_sp_13982
---------- ---------- ---------- ---------- ---------- ----------
---------- ------MFAK KRGYSVDPNQ ELIAYGAGNF VGSFFSCFPI CNALARTAVQ
ENLATQLCSV VVVALVLMVL LFIAPLFFHL PKAILAAVVI ANLIGLLKQF SRLRALWGIY
KPDAFVWFFS CFGVILLGVD KGLGVGVICA L---------
>Stylophora_pistillata_SLC26beta
GIIQIIMGAV KMGFLATFLS EPLISGFTTG SAVLVVNSQL KHILGGVLCL IVLGLPPLSV
PVIAVVIFAT NISISKMFAK KRGYSVDPNQ ELIAYGAGNL AGSFFSCFPI CNALARTAVQ
ENLATQLCSV VVVALVLMVL LFIAPLFFHL PKAILAAVVI ANLIGLLKQF SRLRALWGIY
KPDAFVWFFS CFGVILLGVD KGLGVGVICA LDCSSFTYID
>Corallium_rubrum_Contig_25677|m.26504
GIIQAAMGLI HLGFVTVYLS RPLVSGFTTG AAFIVFTSQV KYFFGGLICI LILGLPGLKA
PLIAVVAATT NLSLVKLFAQ KHEYETDSNQ ELFGYGCVNI ISSFFSCFVS SGSLSRSVVQ
EGIGTQVTSF ISSVIILLVL LFIAPLFEPL PKPVLAAIVV VSLRRLFMQF KQLKSIGKIS
QVDALIWFTS CFAVVLLGIV MGLILGITVT LDVFAVTMVD
>Acropora_digitifera_4974
GLFQFVMGLL NLGFVAVYLS DPIISGFTTG AAVLVFTSQI KHILGGVVCI TLLGIRAPSA
PVIAIVIFAT NVSLAKTFAR RNNYVIDSNQ ELLACGSANI MGSFFSCFPV SASLSRSAIQ
ESIATQLCTI PVVAVVILVL LFIARLFYHL PKAILAAVII VALKGLFLQF SRLKQLWKIC
KPDAVVWFSA WLGVILLGID VGLGVGVVMA LDASTFNFID
>Acropora_millepora_5827
GLFQFVMGVL KLGFVAVYLS DPIISGFTTG AAVLVFTSQI KHILGGVVCI TLLGIRAPSA
PVIAIVIFAT NVSLAKTFAR RNNYVIDSNQ ELLACGSANI MGSFFSCFPV SASLSRSAIQ
ESIATQLCTI PVVAVVILVL LFIARLFYHL PKAILAAVII VALKGLFLQF SRLKQLWKIC
KPDAVVWFSA WLGVILLGID VGLGVGVVMA LDASTFNFID
>Favia_sp_39390
GLFQFVMGVL KLGFVAVYLS DPIISGFTTG AAILVFTSQV KHILGGAICI LLLGLRVPSA
PVIAVVIFAT NVSLAKTFAT RNNYVIDSNQ ELIACGSANI FGSFFSCFPV SGSLSRSVIQ
ESIATQLCSI PVVLIIILVL LFIAPLFFHL PKAILAAIVV VALKGLFRQF SRLVQLWRIC
KCDAVVWFVS WLGVVLLGID IGLGVGVIMA LDASTFNFID
>Porites_australiensis_7954
GIFQFVMGLL KLGFVAVYLS DPIISGFTTG AAILVFTSQV KHLLGGIICI LLLGLRVPSA
PVIAIVIFAT NVSLAKVFAK RNNYVVDSNQ ELIACGSANI LGSFFSCFPV SGSLSRSVIQ
ESIATQLCTI PVVIVIILVL LFIAPLFYHL PKAILAAVVV VALKGLFRQF TRLVQLWKIC
KLDAVVWFAA WLGVVLLGID VGLGVGVVVA LDASTFNFID
>Montastraea_cavernosa_65176
GLFQFVMGLL KLGFVAVYLS DPIISGFTTG AAILVFTSQV KHILGGVICI LLLGLRVPSA
PVIAVVIFAT NVSLAKTFAK RNNYVIDSNQ ELIACGSANI FGSFFSCFPV SGSLSRSVIQ
ESIATQLCSI PVVLIIILVL LFIAPLFYHL PKAILAAVVV VALKGLFRQF SRLVQLWRIC
KRDAVVWFAT WLGVVLLGID IGLGVGVVMA L---------
>Seriatopora_sp_12625
---------- ---------- ---------- ---------- ---------- ---GLRVPSA
PVISIVIFAT NISLAKTFAK RNNYVIDSNQ ELIACGSANV LGSFFSCFPV SGSLSRSVIQ
ESIATQLCSI PVVVVIILVL LFIAPLFYHL PKAILASVVV VALKGLFRQF SRLVQLWRMC
KPDAVVWFAA WLGVVLLGID IGLGVGVIMA L---------
>SLC26alpha
GLLQLLMGLF KLGFVAVYLS DPIISGFTTG AAILVFTSQV KHILGGVVCI LIILGLRVPSA
PVISIVIFAT NISLAKTFAK RNNYVIDSNQ ELIACGSANV LGSFFSCFPV SGSLSRSVIQ
ESIATQLCSI PVVVVIIMVL LFIAPLFYHL PKAILAAVVV VALKGLFRQF SRLVQLWRMC
```

KPDAVVWFAA WLGVVLLGID IGLGVGVIMA LDASTFNFID

SLC4

```
>Nematostella_vectensis_11142
LLNVHKTKIE DIADLPSIQQ SHIAMSRLRH ATNLGRTLQG ---THLVVLI LSPGRTFATL
GIRDDFMRRM PHYLSDFKDG VVLVSTVIFL YFACILPSVA FGVLNSKNTK GKMDVQKVLV
SQVFGGLFFS LLGGQPLIIL LTTAPLALYK HR-------- ---------R ISLRKE----
--------LL SLGTVLLGVT LYNFKKSPFL DANKREALAD YALPVAVLFF --------VV
LCSSTHIQEL KKGSAFHLDL LVVAIINGIL SAFGLPWIHG ALPHSPLHVR ALADVEERVD
QGVFDIIVKV RETRLTTLIA HILIGLSLLM LPLDYIPTAV LDGLASSHNA QVF---ALLE
NN--CFLLQS AYPPNHYIRR VPQRKMHAYT GMQMLQLGIL CGFGFSKMVF PVLLLLLLPI
R
>Montastraea_cavernosa_106688
LLGLHETSLQ GIGELPSVQQ SHVAIAQLRH PVNLGRTLEE ---THLVVLV LAPGRTFATL
GLIRDLKRRW PHYLSDFKDG IRLISTTLFL YFACLLPSIA FGVLNSRNTN GQIDVQKVII
SQAVGGMLFA LFGGQPLIVM LTTAPLALYG LWNSF----- ---------- ----------
---------- ---------- ---------- ---------- ---------- ----------
---------- ---------- ---------- ---------- ---------- ----------
---------- ---------- ---------- ---------- ---------- ----------
---------- ---------- ---------- ---------- ---------- ----------
-
>Acropora_digitifera_14452
LLDLQETSLQ GIGELPTVQQ SHVAIARLKH PVNLGRSLEE ---THLVVLV LAPGKTFATL
GLMRDLKRRL PHYLSDYKDG TRVVSTTLFL YFACLLPCIA FGVLNGRNTE GQIDVQKVII
SQAFGGILFA LFGGQPLIVL LTTAPLALYG LWNAFFLFIY STFGLSQIMK WSTRSTEEIF
ALFISLAFVI SLGTVWIGLA LYNFRKSPFL DAGKREALAD YALVVAVLAM LFFMDQNISS
AMVNNPGNRL KKGSAYHWDL LVVGFVNAFL SIFGLPWVHA ALPHSPLHVR ALADVEERVD
RGVFEIIVKV RETRLTGLLS SILIALSLLM LPLKLVPTPV LDGLFLFMAI TALDGNQMFE
RALLLITEQA AYPPNHYIRH VPQRKMHIYT ALQFLQLGIL CGFGFAKMVF PVLLMLILPI
R
>Acropora_millepora_2965
LLDLQETSLQ GIGELPSVQQ SHVAIARLKH PVNLGRSLEE ---THLVVLV LAPGKTFATL
GLMRDLKRRL PHYLSDYKDG TRVVSTTLFL YFACLLPCIA FGVLNSRNTE GQIDVQKVII
SQAFGGILFA LFGGQPLIVL LTTAPLALYG LWNAFFLFIY STFGLSQIMK WSTRSTEEIF
ALFISLAFVI SLGTVWIGLA LYNFRKSPFL DAGKREALAD YALVVAVLAM LFFMDQNISS
AMVNNPGNRL KKGSAYHWDL LVVGFVNAFL SIFGLPWVHA ALPHSPLHVR ALADVEERVD
RGVFEIIVKV RETRLTGLLS SILIALSLLM LPLKLVPTPV LDGLFLFMAI TALDGNQMFE
RALLLITEQA AYPPNHYIRH VPQRKMHIYT ALQFLQLGIL CGFGFAKMVF PVLLMLILPI
R
>Favia_sp_24777
---------- ---------- ---------- ---------- ---------- ----------
---------- ---------- ---------- ---------- ---------- ----------
---------- ---------- ---------- ---------- ---------- ----------
---------- ---------- ---------- ---------- ---------- ----------
---------- ---------- -------SFL SLFGLPWVHA ALPHSPFHVR ALADVEERVD
RGVFEIIVKV RETRLSGLLS SSMIALSLLM LPLTLIPTSV LDGLFLFMAI TSLDGNQMFE
RALLLVTEQA AYPPNHYIRH VPQRKMHLYT ALQFLMLGVL CGFGFAKMVF PVLLMLILPI
R
>Seriatopora_sp_11670
---------- ---------- ---------- ---------- ---------- ----------
---------- ---------- ---------- ---------- ---------- ----------
---------- ---------- ---------- ---------- MNIESELFIK FKAXSTEEIF
```

```
ALFISLAFVT SLGTVWIGVA LYNFRKSPFL DAGKREALAD YALVVAVLVM ----------
---------- KKGSAYHWDL LVVAVVNGFL SIFGLPWVHA ALPHSPFHVR ALADVEERVD
RGVFEIIVKV RETRLTGFLS STLIALSLLM LPLTLIPTPV LDGLFLFMAM TSLYGNQMFE
RALLLVTEQA AYPPNHYIRH VPQRKMHLYT ALQFLQLGVL CGFGFAKMVF PVLLMFILPI
R
>Stylophora_pistillata_SLC4epsilon
LLGLQETSLQ GIGELPSVQQ SHVAFAQLRH PVNLGRTLEE ---THLVVLV LAPGRTFATL
GVIRDFRRRW PHYLSDFKDG IRVISTTLFL YFACILPSIA FGVLNSRNTS GKIDVLKVII
SQSVGGILFA LFGGQPLIVL LTTAPLALYG LWNSFFLFIY STFGLSQIMK WSTRSTEEIF
ALFISLAFVT SLGTVWIGVA LYNFRKSPFL DAGKREALAD YALVVAVLVM LFFMDQNISS
AMVNNPGNRL KKGSAYHWDL LVVAVVNGFL SIFGLPWVHA ALPHSPFHVR ALADVEERVD
RGVFEIIVKV RETRLTGFLS STLIALSLLM LPLTLIPTPV LDGLFLFMAM TSLYGNQMFE
RALLLVTEQA AYPPNHYIRH VPQRKMHLYT ALQFLQLGIL CGFGFAKMVF PVLLMFILPI
R
>Porites_australiensis_9087
LLDMNASNLT QIGELDFLKK PVTAFVRLEN AHILGDLTEV PVPTRFIFVM LGPGRAIGTL
GLINDIKRRA PWYWSDFKDA LNCIASVIFI YFACLTPIIT FGGLMGTKTG KNMAAMEQIL
AGGIGGVLFS LFGGQPLIIL GATGPMLVFG IWTMLYCFIL VITDASAFVR YFTRFTEESF
ATLIALIFIL SVGTFALAIS LKGFRNSSYF PTRVRAVISD FAILIAIIIM LVFMDQQITA
LIVNRREHKL KKGAGYHLDL LLVAIIIGIC SLLGLPWVVA ATVLSVGHVQ SLFVESQCTA
PGEKVQFLGV REQRVTGFFI FLLIGLTVFL APLKYVPMPV LFGLFFYMGF SALKGLQFYE
RLKIIFMPIK HQPDLMYLRQ VPLKRIHIFT FIQLFCLAVL WAIKSTALIF PVMVLMLVAV
R
>Acropora_digitifera_15019
LLDMNATNVT QIGELDFLKK PVTAFVRLEN ACVLGNITEV PVPTRFIFLM LGPGRAIATL
GLIADMKRRA PWYWSDFKDA LNCIASIIFI YFACLTPIIT FGGVMGTKTG KNMAAMEQIL
AGGIGGVLFS LFGGQPLIIL GATGPMLVFG IWTMLYCFIL VITDASAFVR YFTRFTEESF
ATLIALIFIL SLGTFALALS LKGFRTSPYF PTKVRAVVSD FAILISIMVM LVFMDQQITA
LIVNRREHKL KKGAGYHLDL LLVAIIIGIC SLLGLPWVVA ATVLSVGHVQ SLFVESQCTA
PGEKTQFLGV REQRVTGTFI FILIGLTVFI APLKYLPMPV LFGLFFYMGF SALKGLQFFE
RLKIIFMPVK HQPDLMYLRQ VPLNRIHIFT FIQLFCLAVL WAIKSTALIF PVMVLMLVAV
R
>Acropora_millepora_1583
LLDMNATNVT QIGELDFLKK PVTAFVRLEN ACVLGNITEV PVPTRFIFLM LGPGRAIATL
GLIADMKRRA PWYWSDFKDA LNCIASIIFI YFACLTPIIT FGGVMGTKTG KNMAAMEQIL
AGGIGGVLFS LFGGQPLIIL GATGPMLVFG IWTMLYCFIL VITDASAFVR YFTRFTEESF
ATLIALIFIL SLGTFALALS LKGFRTSPYF PTKVRAVVSD FAILISIMVM LVFMDQQITA
LIVNRREHKL KKGAGYHLDL LLVAIIIGIC SLLGLPWVVA ATVLSVGHVQ SLFVESQCTA
PGEKTQFLGV REQRVTGTFI FILIGLTVFI APLKYLPMPV LFGLFFYMGF SALKGLQFFE
RLKIIFMPVK HQPDLMYLRQ VPLNRIHIFT FIQLFCLAVL WAIKSTALIF PVMVLMLVAV
R
>Seriatopora_sp_9219
LLDMNATNLS QIGEMDIIKK PVTAFVRLEN ATLLGDLTEV PVPSKFIFFM LGPGRAIGTL
GLIADIRRRA PWYWSDFKDA LNCIASIIFI YFACLTPIIT FGGLMGTKTG KNMAAMEQIL
AGGIGGVLFS LFSGQPLIIL GATGPMLVFG IWTMLYCFVL VVTDASALVR YFTRFTEESF
ATLIALIFIL SLGTFFLAVS LKEFRSSSYF PTKVRSVISD FAILISIMVM LVFMDQQITA
LIVNRREHKL KKGAGYHLDL LLVAIIIGIC SLLGLPWVVA ATVLSVGHVQ SLFVESQCTA
PGEKAQFLGV REQRITGTFI FILIGLTVFI APLK------ ---------- ----------
---------- ---------- ---------- ---------- ---------- ----------
-
>Stylophora_pistillata_SLC4delta
LLDMNATNLS QIGEMDIIKK PVTAFVRLEN ASLLGDLTEV PVPSKFIFLM LGPGRAIGTL
GLIADIRRRA PWYWSDFKDA LNCIASIIFI YFACLTPIIT FGGLMGTKTG KNMAAMEQIL
```

```
AGGIGGVLFS LFGGQPLIIL GATGPMLVFG IWTMLYCFVL VVTDASALVR YFTRFTEESF
ATLIALIFIL SLGTFFLAVT LKEFRSSSYF PTKVRSVISD FAILISIMVM LVFMDQQITA
LIVNRREHKL KKGAGYHLDL LLVALIIGIC SLLGLPWVVA ATVLSVGHVQ SLFVESQCTA
PGEKAQFLGV REQRITGTFI FILIGLTVFI APLKFVPMPV LFGLFFYMGF SALRGLQFFE
RLKIMFMPVK HQPDLMFLRQ VPLKRIHIFT LIQLLCLGIL WLIKSTALIF PVMVLMLVAV
R
>Favia_sp_53015
LLDMNASNLP QIGELDFLKK PVTAFVRLEN ASLLGDLTEV PVPTRFIFLM IGPGRAIATL
GLLADIRRRA PWYWSDFKDA LNCIASIIFI YFACLTPIIT FGGLMGTKTG KNMAAMEQIL
AGGIGGVLFS LFGGQPLIIL GATGPMLVFG IWTMLYCFVL VVTDASALVR YFTRFTEESF
ATLIALIFIL ALGTFFLAIS LKGFRTSSYF PTKVRAVVSD FAILISIMIM LVFMDQQITA
LIVNRREHKL KKGAGYHLDL LLVAIIIGIC SLLGLPWVVA ATVLSVGHVQ SLFVESQCTA
PGEKAKFLGV REQRVTGTMI FILIGLTVFM SPLKFVPMPV LFGLFFYMGV SALRGLQFFE
RLKIIFMPIK HQPDLMYLRQ VPVNRIHIFT LIQLFCLGVL WLIKSTALIF PVMVLMLVAV
R
>Montastraea_cavernosa_110116
LLDMNASNLP QIGELDFLKK PVTAFVRLEN ASLLGDLTEV PVPTRFIFFM LGPGRAIATL
GLIADMRRRA PWYLSDFKDA LNCIASIIFI YFACLTPIIT FGGLMGTKTG KNMAAMEQIL
AGGIGGVLFS LFGGQPLIIL GATGPMLVFG IWTMLYCFVL VITDASALVR YFTRFTEESF
ATLIALIFIL ALGTFFLAIT LKGFRTSSYF PTKVRAVVSD FAILISIMIM LVFMDQQITA
LIVNRREHKL KKGAGYHLDL LLVAIIIGIC SLLGLPWVVA ATVLSVGHVQ SLFVESSCTA
PGEKAKFLGV REQRITGTMI FILIGLTVFM SP-------- ---------- ----------
---------- ---------- ---------- ---------- ---------- ----------
-
>Hcoerulea_m.167661
LLDLNASTLP QIGETNFLSK HINAFIRLED AQNLGDIMEV PIPIRFIFLM LGPGRSIATL
GLICDIKRRA KVYKSDVVDA LNCLASILFI YFACLAPIVT FGGVMQGKTD NYMGAMEQLL
ACCIGGVLFS LFSGQPLTIL GATGPMLVFG LWTSFFCLLL VVSDASALVR YFTRFTEESF
ATLIGLIFIL TFGTFFLVVK LKAFKTSRFF PTKVRKVVSD FAVIISVMIM LVFMDQQITA
VIVNKREHKL RKGAGYHLDL TIVAVAIAIN SMLGLPWVVA ATVLSLSHVQ SLFVESQCTA
PGERPKFLGV REQRVTGTVV FTLVGCTVFL GKLKYIPMPV LYGLFIYMGV SALRGVQFFE
RLKIIFMPIK HQPDRVYLRK VRIRRIHIFT AIQVFCLAFL WAVKTTAITF PLMVLALVAV
R
>Corallium_rubrum_Contig_27997|m.28609
LLDLNASTIT QIGETDFLPK SVNAFVRLED AQLLGDLMEV PIPTRFLFFM LGPGRAISTL
GLICDIKRKA KVYKSDFIDA LNCLASILFI YFACLAPIVT FGGVMQIKTE NYMGAMEQLL
AASIGGVLFS LFSGQPLTIL GATGPMLVFG LWTTFFCLVL VVTDASALVR YFTRFTEESF
ATLIGLIFIL TLGTFFLVIK LKAFKTSRFF PTKVRKVISD FAVIISVMIM LVFMDQQITG
VIVNKREHKL KKGAGYHLDL VVVAVSIGIC SILGLPWVVA ATVLSLAHVQ SLFVESTCTA
PGERPKFLGV REQRVTGTVV FILVGLTVFL GKLKYIPMPV LYGLFIYMGV SALKGVQFFE
RIKIMFMPVK HQPDRVYIRK VRISKIHIFT AIQCSCLIIL WAIKTTAITF PLMVLALVLV
R
>Gorgonia_ventalina_58110
LLDLNANTIP QIGEADFLAK PVNAFIRLED AVVMGDLMEV PIPTRFLFFM IGPGRAIATL
GLVMDIKRKV KCYKSDFVDA LNCLASVLFI YFACLAPIVT FGGVMETKTN KYMGAMEQLL
AASLGGVLFS LFAGQPLTIL GATGPMLVFG LWTTFFCLLL VVTDASALVR YFTRFTEESF
ATLIGLIFVL TFGTFFLVIK LKSFKTSRFF PTRVRKVVSD FAVIISVMIM LVFMDQQITA
VIVNKREHKL KKGAGYHLDL TVVAIGIGIC SILGLPWVVA ATVLSLAHVQ SLFVESTCTA
PGERPKFLGV REQRVTGTLV FILVGLTVFL GKLKYIPMPV LYGLFIYMGV SALKGVQFFE
RIKIFFMPVK HQPDRVYLRK VRIRRIHIFT AIQVICLAIL WAIKATGITF PLMVLALVAV
R
>Nematostella_vectensis_6703
LLDLDEDQLP SIGGHKHLSA PEGAFVRLAR GCRLRNLAEV NIPVRFLFIL LGPGRCVATL
```

GLLRDVKRRF PYYKSDMTDS FNCVMATILV YVACLAPAIS FGGLLYKKTK GWMGVAEMIV
STALSGVIFA LFAGQPLIII GATGPLLLFG IWVMLICWAL VAMEGCFLIR YFTRFTEEIF
ACMISLIFIL FFGTFFVAHI IRDVRHSRFL NHTLRRVISD FGVLIAIVAM LVFMEVEFCN
VILDKKDNQL KKGPGYNLDL FVVGFLMGMC SVLGLPWMCA TPVHTVSHLH ALMVHSTNHA
PGEHPQLLEV KEQRVTNIII HVLIGLTMLL APIRLTPVVV LFGVFVHLGF SSLSHLQFVE
RFKLLFVSPN HHPDRRYVRS VSTGKMNAFT LVQVVCLLFL VAIKVTAPFF PFFVICLVPL
R
>Seriatopora_sp_6433
---------- ---------- ---------- ---------- ---------- ----------
GLIRDAKRRF PLYLSDFKDA LDCLPTIIFE YFACLAPAIA FGGLLSEKTN AWMGVSEMIF
ATALSGVLFG LFAGQPLIII GATGPLLVFG IWVMVVCVII VAVEGCFLIK YFTRFTEEIF
TLMISIIFVL MLGTFFVAHT LRKLRHSHFF GPIARRIVSD FGVFIAIVSM LIFMEIEFTG
IILDKKEHKL KKGVGYNLDL FVLGVLVGLC SVLGLPWMCA TPVHTLSHFH ALTVLSTNNA
PGEHPHLVKV REQRLTNIVI HLLIGFTVLL SPMRLIPIAV LFGVFLFLGV SSLSHIQLVQ
RIKLLFIPAS HHPVEKFVMN VKTKKMHLFT IVQVCCVCAL VAMKLTAPAF PFFIICMIPL
R
>Stylophora_pistillata_SLC4alpha
LLDLNRDTLP SIGAHNGLSC TVSAFIRLAK GCELSNLAEV QIPIRFIFIL VGPGRSFATL
GLIRDVKRRF PFYLSDFKDA LDCLPTIIFV YFACLAPTIA FGGLLSEKTN AWMGVSEMIF
ATALSGVLFG LFAGQPLIII GATGPLLIFG IWVMVICFII VAVEGCFLIK YFTRFTEEIF
ALVISIIFVL MLGTFFVAYT LRKLHHSHFF GPKARRIVSD FGVFIAIVSM LIFMEIEFTG
IILDKKEHKL KKGVGYNLDL FVLGLLVGLC SVLGLPWMCA TPVHTLSHFH ALTVLSTNHA
PGEHPRLVQV REQRLTNIVI HLLIGFTVLL SPMRLIPIAV LFGVFLFLGV SSLSHIQLVK
RIKLLFIPAS HHPVEKFVTN VKTRKMHLFT IVQVCCVCAL VALKLTAPAF PFLIICMIPL
R
>Corallium_rubrum_Contig_41247|m.41370
LLDLEQFDLS NIGTMDELEK PAMAFVRLAK GCHLGNLTEL PLPVRFIFVL LGPGRSIATL
GLINDFRRKR PHYWSDFVDA FKCLVAFIFV YFAAIAPCIT YGGLLSKKTE GWIGLSESIL
ATALGGILFG LFSGQPLMIV GVTGPVLVFG LWIVVICTLV IAFEGCFLVK YFTRFTEEIF
ACLISFIFIL LIGTVLLTVR LRKFRHSHFI GRKGRRTVSD FGMAISIIAM LLFMETELTG
IVLNKKENNL RKGAGYNLDL LVVGLVCGVC SFMGLPWICA GPVRSVSHKN ALTVMSTSHA
PGERAHVVRV IEQRVTNIVI HIFIGLSVLL APLKIIPIAV LFGVFVYLGM TSLSGNQFVI
RFVMFFMPTK HYPDFRFVRK VSGGKIHLYT LFQLLPLFIL IAVKMTAPLF PLLIICLVPL
R
>Hcoerulea_m.134026
---------- ---------- ---------- ---------- ---------- ----------
GLIKDVRRKY PHYLSDFVDA FKCLVAFIFV YFAAIASCIT YGGLLSKKTD GWLGLSESIL
ATALGGILFG LFSGQPLMIV GVTGPVLVFG LWIVVICTLV VAFDGCFLVK YFTRFTEEIF
ACLISFIFIL LVGTVMLTVG LRKFRHSHFI GRKGRRTVSD FGMAISIIAM LLFMETELTG
IVMNKKENNL KKGAGYNLDL LMVGLVCGIC SIMGLPWICV GPVRSVSHKN ALTVMSTSHA
PGERPHLVKV IEQRATNIVI HIFIGLSVFL APLKIIPIAV LFGVFVYLGT TSLSGNQLVI
RVVMFFMPSK HYPDYRFVRK VSGYKIYLFT VIQLFPLAIL IGVKMTAPLF PLLIICLVPL
R
>Nematostella_vectensis_1693
LLDLEQFDLQ SIGTLDCLKN PVTAFVRLAK GCYLGNVTEV AIPVRFLFVM LGPGRSIATL
GLIQDIKHRS KTYISDFKDG FNCLLVTVFL YFAVFAPNVA FGGLLAEKTD QWLGVSEVIF
ASCFCGLLFA LFSGQPLIII GATGPVMVFG FWVLVILFLV VAFEGCFLVR YFTRFTEELF
ACLISLIFIL MFGTYFVAFY MRKFRNSHFF GKKARRLVSD FGIAIAMITM LLFMETELTG
VLINKKENCL QKRPGYNMDL LLMGVITLIC SLFGLPWMCP ATVRSVSHFN ALSVWSTSHA
PGEKPYLVEV REQRITNILI HVLTGMSILL APLHHVPVPV LFGVLLYLGL SSLSNLQLIN
RLIMMLMPPK HHPDVRYVRK VPTAKIHGYT LIQLACLLVL MAVKLTAPSF PFFIICLIPV
R
>Seriatopora_sp_14071

LLDLEKFDLP SIGTLDELQY PVLAFVRLAK GCPL-NITEV SIPVRFMFVL LGPGRSVATL
GLVKDVKRRA KVYLSDFKDG INCLLTSIFL YFSVFAPNVA FGSLLAKKTD EWLGVSEVIL
ATCMCGVLFG LFAGQPLIII GATGPVLVFG FWVMMILFGV VALEGCFLVR YFTRFTEEIF
ACLISAIFIM VLGTFFVAFY LRKFRTSYFF GKKVCNDLN- ---------- ----------
---------- ---------- ---------- ---------- ---------- ----------
---------- ---------- ---------- ---------- ---------- ----------
---------- ---------- ---------- ---------- ---------- ----------
-
>Acropora_digitifera_13594
LLDLEKFDLP SIGTLDELQQ SVLAFVRLAK GCHL-NITEV SIPVRFLFVL LGPGRSVATL
GLIADIKRRT KFYLSDFRDG FNCLLTSIFL YFSVFAPNVA FGSILHKKTA GWLGVSEVIY
ATCLCGVLFG LFAGQPLIII GATGPVLVFG FWVMIILFGV VALEGCFLVR YFTRFTEEIF
ACLISVIFIL LLGTFFVAFY LRKFRTSYFF GKRARRLVSD FGIVIAMASM LLFMETELTG
VLLNKKENKL TKGAGFNLDL CVMGVLSFIC SLMGLPWMCA ATVRSVSHLQ SLTVWSTSHA
PGVKPHITEV KEQRITNICI HILTG----- ---------- ---------- ----------
---------- ---------- ---------- ---------- ---------- ----------
-
>Acropora_millepora_327
LLDLEKFDLP SIGTLDELQQ SVLAFVRLAK GCHL-NITEV SIPVRFLFVL LGPGRSVATL
GLIEDIKRRT KFYLSDFRDG FNCLLTSIFL YFSVFAPNVA FGSILHKKTA GWLGVSEVIY
ATCLCGVLFG LFAGQPLIII GATGPVLVFG FWVMIILFGV VALEGCFLVR YFTRFTEEIF
ACLISVIFIL LLGTFFVAFY LRKFRTSYFF GKRARRLVSD FGIVIAMASM LLFMETELTG
VLLNKKENKL TKGAGFNLDL CVMGVLSFIC SLMGLPWMCA ATVRSVSHLQ SLTVWSTSHA
PGVKPHITEV KEQRITNICI HILTGISILL APLNRIPVPV MFGVLLYLGV CSLSGIQLVD
RFIMMFMPHK YHPDVQYVRK VNFLKIHCYT GIQLICLALL IVVKVTAPSF PFFIICLIPL
R
>Porites_australiensis_10591
LLDLEKFDLP SIGTLDELEH SVLAFVRLAK GCDL-NITEV SIPVRFLFVL LGPGRSVATL
GLIQDVKRRT KVYLSDFTDG FNCLLTSIFL YFSVFAPNVA FGSILAKKTD GWLGVSEVIF
ATCLCGVLFG LFAGQPLIII GTTGPVLVFG FWVMIILFGV VALEGCFLVR YFTRFTEEIF
ATLISAIFIL VLGTFFVAFY MRKLRTSYFF GKRARRLVSD FGIVIAMASM LLFMETELTG
VLINKKENKL LKGAGFNLDL CVMGLLSFIC SLMGLPWMCA ATVRSVSHLQ SLTIWSTSHA
PGVKPHIVEV KEQRVTNIAI HVLTGISILL APLNRIPVPV MFGVLLYLGV CSLSGIQLID
RLIMMFMPPK YHPDVQYVRK VKTKQIYFYT IIQLICLALL VAVKLTASSF PFFIICLIPL
R
>Stylophora_pistillata_SLC4beta
LLDLEKFDLP SIGTLDELQY PVVAFVRLAK GCPL-NITEV SIPVRFMFVL LGPGRSVATL
GLVEDIKRRA KVYLSDFKDG LNCLLTSIFL YFSVFAPNVA FGSLLDKKTE GWLGVSEVIL
ATCMCGILFG LLAGQPLIII GATGPVLVFG FWVMMILFGV VALEGCFLVR YFTRFTEEIF
ACLISAIFIM VLGTFFVAFY LRKFRTSYFF GKKARRLVSD FGIVIAMACM LLFMETELTG
VLLNKKENKL LKGAGFNLDL IVMGCLSFVC SMMGLPWMCA ATVRSVSHLN ALSIWSTSQA
PGVKPHLVEV KEQRVTNIAI HVLTGVSILL APLHRIPVAV MFGVLFYLGV CSLSGIQLVD
RIIMMFMPPK YHPDVQYVRK VKTRKIHSYT IIQVVCLILL IVVKLNAPSF PFFIICLIPL
R
>Favia_sp_54727
---------- ---------- ---------- ---------- ---------- ----------
---------- ---------- ---------- ---------- ---------- ----------
----CGIFFG LLAGQPLIII GVTGPVLVFG FWVMLILFGV VAMEGCFLVR YFTRFTEEIF
ACLISAIFIM VLGTFLVAFY LRKFRTSYFF GKRARRLVSD FGIVIAMALM LLFMETELTG
VLLNKKENKL CKGAGFNLDL CVMGLLSFIC SLMGLPWMCA ATVRSVSHLN ALSVWSTSHA
PGVKPHLIEV KEQRVTNIAI HVLTGLSILL APLHLIPVPV MFGVLLYLGV CSLAGIQLVD
RLIMMFMPPK YHPDVQYVRK VSTKQIHIYT AIQLVCFALL IVVKLTAPSF PFFIICLIPL
R

>Montastraea_cavernosa_107903
LLDLEKFDLP SIGTLDELQQ PVLAFVRLAK GCPL-NITEV SIPVRFLFVL LGPGRSVATL
GLGLDIKRRA KFYLSDFKDG FNCLLTSIFL YFSVFAPNIA FGSILDKKTD GWLGVSEVIF
ATCLCGILFG LLAGQPLIII GVTGPVLVFG FWVMLILFGV VAMEGCFLVR YFTRFTEEIF
ACLISAIFIM VLGTFLVAFY LRKFRTSYFF GKKARRLVSD FGIVIAMASM LLFMETELTG
VLLNKKENKL TKGPGFNLDL CVMGLLSFIC SLMGLPWMCA ATVRSVSHLN ALSIWSTSHA
PGVKPHLIEV KEQRVTNIAI HALTGLSILL APLHLIPVPV MFGVLLYLGV LSLAGIQLVD
RLIMMFMPPK YHPDVQYVRK VKTKQIHIYT VIQLICLVLL TAIKLTAPSF PFFIICLIPL
R
>Seriatopora_sp_21473
LLDLEKFDLP NIGTLDELQD PVMAFVRLAK GVYLEEVSEV SIPVRFLFIM LGPGRSVATL
GLIQDVKRRG KVYVDDFRDG FNTLLASLFL YFALFATNIA FGGLFEDKTE GWLGLTEVIF
AACACSILFG LFGGQPIIII GATGPMLVFG FWVMIILFGV VALEGCFLIK YFTRFTEDIF
ELLISAIFIL VLGTFFIAFY MRKLRTSHFF GKRARRLVSD SGIVIAMFIM ----------
---------- ---------- ---------- ---------- ---------- ----------
---------- ---------- ---------- ---------- ---------- ----------
---------- ---------- ---------- ---------- ---------- ----------
-
>Stylophora_pistillata_SLC4gamma
LLDLEKFDLP NIGTLDELQD PVMAFVRLAK GVHLEEMSEV SIPVRFLFIM LGPGRSVATL
GLIQDVKRRG KVYLSDFRDG FNTLLASLFL YFALFATNIA FGGLFEDKTE GWLGLTEVIF
AACACSILFG LFGGQPIMII GATGPMLVFG FWVMIILFGV VALEGCFLIK YFTRFTEDIF
ELLISAIFIL VLGTFFIAFY MRKLRTSHFF GKRARRLVSD SGIVIAMFIM LLFMETEMTG
VLLSKKEHKL AKGPGYNMDM CVVGMLAFGC SLLGLPWMCA DTVRSASHVN ALSIWSTSHA
PGEKPHLIEV KEQRISNIIV HVLTGLSILL APLNVIPIPI FFGVLLYLGI VSMYGLQMVD
RFIMMFMPRK HHPDVGYVRK VRTGKIHCYT VIQVLALAFL VGIKLSAPSF PFFIICLIPL
R
>Porites_australiensis_44499
LLDLEKSDLP HIGTLEELDR SVLAFVRLSK GCNLGQVTEV SIPVRFLFVM LGPGRSIATL
GLFQDIKRRG KFYMSDFYDG FNSLLASLFL YFALFATNIA FGGLLENKTE KFLGVTEVII
SASICSIILG LFAGQPMMII GATGPMLVFG FWVMIILFGV VALEGCFLIK YFTRFTEDIF
ELLISAIFIL VIGTFFLAYY MHKFRTSHFF GKRGRRIVSD SAVVIAMVSM LLFMETEMTG
VLLSKKEHKL HKGPGYNMDL VVVGVLAFGC SLMGLPWMCA DTVRSASHVN ALSIWSTSHA
PGEKPRLLEV KEQRVTNIII HVLTGLSIFL APIILIPIPI FFGVLLHLGI LSLSGPQMVE
RFVMMFMPRK HHPDVGYVRK VPTKKIHMYT IIQTLAWAFL FGIKLSAPSF PFFIICLIPL
R
>Acropora_digitifera_4890
LLDLQRFDLQ SIGTLDDLED SVLAFVRLSK GCNLGQVSEV SIPVRFLFVM LGPGRSIATL
GLFQDIKRRG KFYLSDFSDG FNSLFASLFL YFTLFATNIA FGGLLEDKTE GWLGVTEVIV
SASACSIILG LFAGQPMMII GATGPILVFG FWVMIILFGV VALEGCFLIK YFTRFTEDIF
ELLISAIFIL VLGTFFVAFY MRKFRTSHFF GKRSRRLVSD SSVVVAMVLM LLFMETEMTG
VLLSKKEHKL QKGPGYNLDL VVVGMLSFGC SLMGLPWMCA DTVRSASHVN ALSIWSTSHA
PGEKPHIIEV KEQRITNILI HVLSGLSILL APLHLIPIPV FFGVLLYLGV CSMYGLQMMD
RFIMLFMPSK HHPDVGYVRK VRTSKIHIFT VIQTLSLAFL VAVKLTAPSF PFFIMCLIPL
R
>Acropora_millepora_4369
LLDLQRFDLQ SIGTLDDLED SVLAFVRLSK GCNLGQVSEV SIPVRFLFVM LGPGRSIATL
GLFQDIKRRG KFYLSDFSDG FNSLFASLFL YFTLFATNIA FGGLLEDKTE GWLGLTEVIV
SASACSIILG LFAGQPMMII GATGPILVFG FWVMIILFGV VALEGCFLIK YFTRFTEDIF
ELLISAIFIL VLGTFFVAFY MRKFRTSHFF GKRSRRLVSD SSVVVAMVLM LLFMETEMTG
VLLSKKEHKL QKGPGYNLDL VVVGMLSFGC SLMGLPWMCA DTVRSASHVN ALSIWSTSHA
PGEKPHIIEV KEQRITNILI HVLSGLSILL APLHLIPIPV FFGVLLYLGV CSMYGLQMMD
RFIMLFMPSK HHPDVGYVRK VRTSKIHIFT VIQTLSLAFL VAVKLTAPSF PFFIMCLIPL

R

CARP 4

```
>Hcoerulea_m.72978
EVDKDGKEVQ NDGDFKAVTF YIAKEAGNIT LGDDTMVEKG QIKFTVHIEG WVWCTKDDAN
CEE-VNEVG- ---------- ---------- ---------- -------
>Corallium_rubrum_Contig_30309|m.30654
EYVD---NIH SFKGFSGEMF TIFNKSGTIE NGVKIKVKGG MVKTDISISN WVFCNSWCTA
SKTETGNILQ FNMTIKGKSS PPKLMTVESK KSFLFRFKKF NGTVFYD
>Nematostella_vectensis_17985
ERDSNGNPVH NFNNFAQLDF YIFAESGSYV WGDETEVKKG SVKFNIRVEG WKFCGEPGFT
CDG-VGSYLD SKICIASKKG SPLL---DSN NCFTARFPKF TNYALYD
>CARP4_AGG36357.1
EKDVDGNDVH SVDSFDDVDF YLFRRAGKVT FGNEFRVEKG TIKFNIRISN WDFCDGSRFD
CEGKIGEFLD LKLKIKSKDS PPKFEVEDGE KKFVFRIPKF KKSVLVD
>Seriatopora_sp_11526
---------- ---------- ---------- ---------- -------ISN WDFCDGSRFD
CEGKIGEFLD LKLKIKSKDS PPKFEVEDGE KKFVFRIPKF KKSVLVD
>Favia_sp_34447
EKDSQGNDVH SVKSFDDVKF YLFRGAGTIT FGNEFAVQSG TVKFNIKISK WDFCDDSPSD
CSGKAGEYLD ISLKVKSKGS P--------- ---------- -------
>Montastraea_cavernosa_2049
EKDADGMDVH FVDSLDDVKF YLFRRGGSIT FGSEFAVQSG TVKFNIKISD WDFCDGTPAD
CEGKTGEYLD LKLKIKSKGS PPKFEFEDGE KKFVFRIPKF NQNVMVD
>Porites_australiensis_31214
EKDADGNDVH SVDSFDDTFF YLFREAGKVK FGTEFNVQAG TVKFNIQISN WNFCKE--TD
CEGKAGEYLD LKLKIKSKGT PPKMEIEDGE MKFKFRIPKF NQNSLID
>Acropora_digitifera_2051
EKDSDGNDVH SVESFKDRPF YLFREDGTVS FGNEFDVQAG TVKFNIKISN WDFCDGSAQD
CEGKAGEYLD VNIKFKSKDT PPKVEIEDGE KKIKFRVPKF DDNVIID
>Acropora_millepora_12217
EKDSDGNDVH SVESFEDRPF YLFREDGTIS FGNEFDVQAG TVKFNIKISN WDFCDGSAQD
CEAKAGEYLD VNIKFKSKDT PPKVEIEDGE KKIKFRVPKF DDNVNID
```

CARP 5

```
>Corallium_rubrum_Contig_30309|m.30654
VMDELSEYVD NITHSFKGFS GEMFAFISAK TFNFTAKINS ANLVTVTIFN KSGTIENGVK
KIKVKGGMVK TDISISNWVF CGNILQFNMT IKGKSSP
>Nematostella_vectensis_15139
DFDSVAEYNA AGRRNFQNFE KLDFKFISCR TFSMEAKIPS TNAVQLFIFK ENGTYEWGDE
AIDVTRGALK FNIEVEDWKF CGEYLEVVIC IKGRKSP
>Hcoerulea_m.102272
EMSMLQELAS DGKHSFNSFA TQSFTFYSVT KVAFTSYLGG GNAIESIIFM EDAILQFENP
DPAIVAGTVK FNVLMSQWNW CGDSVRLTIT MTNQITS
>Porites_australiensis_45651
ELDDIKEKDD NDKHSVDSFK DVQFQFIPAI NVNLTGRLED ADAIMVYLFR QAGSITFGNE
TFQVQPGTVK FNIKISDWDF CGQFLDLSMR VKSKGTP
>Acropora_digitifera_2051
ELDSIEEKDS DGGHSVESFK DRPFSLVAAI NVNLSTKLED SNAIMLYLFR EDGTVSFGNE
TFDVQAGTVK FNIKISNWDF CGEYLDVNIK FKSKDTP
>Acropora_millepora_12217
```

```
ELDSIEEKDS DGGHSVESFE DRPFSLVAAI NVNLSTKLED SNAIMLYLFR EDGTISFGNE
TFDVQAGTVK FNIKISNWDF CGEYLDVNIK FKSKDTP
>Favia_sp_34447
ELDEIEEKDS QGKHSVKSFD DVKFTFIPVI KMNYSVYLPD PDAIIVYLFR GAGTITFGNE
TFAVQSGTVK FNIKISKWDF CGEYLDISLK VKSKGSP
>Montastraea_cavernosa_8912
EVDEIEEKDS QGKHSVKSFD NVKFTFIPVT KMNYSAYLPD PKAIIVYLFR RAGSINFGNE
TFSVQSGTVK FNIKISNWDF CGEYLDLSLK VKSKGSP
>Stylophora_pistillata_16791
ELDEIEEKDA DDKHSVDSFD DVKFTFIRVT TVNLSTYLDN QKAIIVYMFH EPGSVRFGNE
TFAVQSGTVK FNIKISNWNF CGEFLDLSLK IKSKGSP
>Seriatopora_sp_19057
ELDEIEEKDA DDKHSVDSFD DVKFTFIHVT TVNLSTYLDD QKAIIVYLFH EAGSVRFGNE
TFAVQSGTVK FNIK------ ---------- -------
```

CARP 1

```
>Nematostella_vectensis
DFKGLSAEET KQKLEQLVKD LDKDGFVTED EDDNKDGKVS WEEFKEQMAE DEAKFKYADV
NGDGMLDLHE YVTFYHPGDD ERMSAWVIQD TLKKHDTDKD GMISKSEYDK NKDGKLDQTE
IRHWLFPDDD AKEEPAHMIK EADDNKDGKL SMEEILKHSS VF
>Montastraea_cavernosa_6381
EFKGLSPEDA KTKLAQLIKD LNKDGVLTED EDEDKDGKVS WEEFKEQMKE DEEKFKFADE
DGDGKLDLEE YLAFYHPGDN PRMAEFTVED SLKKHDKDKD GQISKLVV-- ----------
---------- ---------- ---------- ---------- --
>Acropora_digitifera_8061
EFKGLSPEEA QTKLIQLIKD QNKDGYLTED EDEDKDGKVS WEEFKEQMKE DEEKFKFADE
DGDGKLDLEE YLAFYHPGDN LRMAGFTIQD SLKKHDKDKD GQISKKEFDK DSSGKLDKDE
MKLWLFPDDD STEEPKTLIK EADEDKDGKL SMDEIKKNYK VF
>Acropora_millepora_17857
---------- ---------- ---------- -DEDKDGKVS WEEFKEQMKE DEEKFKFADE
DGDGKLDLEE YLAFYHPGDN LRMAGFTIQD SLKKHDKDKD GQISKKEFDK DSSGKLDKDE
MKLWLFPDDD STEEPKTLIK EADEDKDGKL SMDEIKKNYK VF
>Favia_sp_39265
---------- ---------- ---------- -STDKDGKVS WEEFKEQMKE DEEKFKFADE
DGDGKLDLEE YLAFYHPGDN PRMAEFTIED SLKKHDKDRD GQISKKEFDK DKSGKLDKKE
MKSWLFPDDD SIEEPKTLIK EADENHDGKL TMDEIMKNYK VF
>Seriatopora_sp_1163
EFKGLSPEDA KTKLAQLIKD LNKDGVLTED EDEDKDGKVS WEEFKEQMKE DEEKFKFADE
DGDGKLDLEE YMAFYHPGDN PRMTEFTIED SLKKHDKDKD GQVSKKEFDK DKNGRLNKEE
MKSWLFPDDD STEEPKTLIK EADEDKDGKL TMDEIMKNYK VF
>Stylophora_pistillata_6464
EFKGLSPEDA KTKLAQLIKD LNKDGMLTED EDEDKDGKVS WEEFKEQMKE DEEKFKFADE
DGDGKLDLEE YMAFYHPGDN PRMTEFTIED SLKKHDKDKD GQISKKEFDK DKNGKLDKEE
MKSWLFPDDD STEEPKTLIK EADEDKDGQL TMDEIMKNYK VF
>Corallium_rubrum_Contig_17442|m.17030
QFDDLEPKIA KLKLREIAKD VNNDQKITAQ EDTNNDDQIS WSEYKEQLKS DERRFDHADM
NGDGKLSREE LIFFLHPEES PLMKDVVAEE NMELLDTNKD GEISLKEFDK NKNGKLSKDE
LMPWFVHDEA S-NEAMHLLS QADTNMDHML SYDEMVDKYE AF
>Gorgonia_ventalina_62208
NFENLDPKIA KNKLREIAKD VNKDGKINAQ EDTNKDEHVS WSEYKERLKS DERRFDKADM
NGDGLLSREE LTFFLHPEES HLMTDVIAEE NLEALDTNND GEVSLKEFDT NKDGKLNKEE
LIPWVVPNEA S-NEAMHLLS QADGNMDHVL SYDEMVNKYE AF
```

```
>Porites_australiensis_10011
EYDDLPPEEA KKRLRILVKD TDKDGFVSEE EDTDKDGKVS WDEYKKMLQN DKRRFDVADK
NKDGGLSKEE FVNFMHPESS PEMGDVHVIE TIEDIDNNKD GYVSLDEFDK NHDGKLDKDE
VKLWILPETD AQEEAQHLIT SADDDKDGKL SEEEIVTNHE TF
>Gorgonia_ventalina_73335
EFDHLSPEEA QRRLKILLKD TDGDSYVSSD EDKNGDGLID WDEFKKMIDR DRKHYDVADT
DKDGKLTATE FGSFLHPESN PEMQALNAQE TLEDMDRNND GKLDLNEFDK NGDGILDSPE
LTAWISPDTD VEEEVKHLIG ESDDDKDGKL SV-------- --
>Hcoerulea_m.114545
EFDHLSPEEA KRRLKLLIAD GDKDSYVTPE EDKDKDGYIS WEEFKKMIER DRKRYNVADS
NKDGKLSPEE YAGLSHPESN PEMQALNAEE TLEDMDKNKD GVLNLDEFDK DHDGKLNNEE
LRAWIAPDTD IEEEVKHLME ESDDDKDGKL SVEEVIKHHE VF
```
